# Cellular and Molecular Immune Response to Chikungunya Virus Infection

**DOI:** 10.3389/fcimb.2018.00345

**Published:** 2018-10-10

**Authors:** Ithallo S. B. Tanabe, Eloiza L. L. Tanabe, Elane C. Santos, Wanessa V. Martins, Isadora M. T. C. Araújo, Maria C. A. Cavalcante, Ana R. V. Lima, Niels O. S. Câmara, Leticia Anderson, Dinar Yunusov, Ênio J. Bassi

**Affiliations:** ^1^IMUNOREG–Grupo de Pesquisa em Regulação da Resposta Imune, Laboratório de Pesquisas em Virologia e Imunologia, Instituto de Ciências Biológicas e da Saúde, Universidade Federal de Alagoas, Maceió, Brazil; ^2^Laboratório de Imunobiologia dos Transplantes, Departamento de Imunologia, Instituto de Ciências Biomédicas, Universidade de São Paulo, São Paulo, Brazil; ^3^Centro Universitário CESMAC, Maceió, Brazil; ^4^Cold Spring Harbor Laboratory, Genome Research Center, Woodbury, NY, United States

**Keywords:** Chikungunya virus, immune response, immunovirology, innate immunity, adaptative immunity, immunological markers, vaccines

## Abstract

Chikungunya virus (CHIKV) is a re-emergent arthropod-borne virus (arbovirus) that causes a disease characterized primarily by fever, rash and severe persistent polyarthralgia. In the last decade, CHIKV has become a serious public health problem causing several outbreaks around the world. Despite the fact that CHIKV has been around since 1952, our knowledge about immunopathology, innate and adaptive immune response involved in this infectious disease is incomplete. In this review, we provide an updated summary of the current knowledge about immune response to CHIKV and about soluble immunological markers associated with the morbidity, prognosis and chronicity of this arbovirus disease. In addition, we discuss the progress in the research of new vaccines for preventing CHIKV infection and the use of monoclonal antibodies as a promising therapeutic strategy.

## Introduction

Chikungunya virus (CHIKV) is an arthropod-borne virus that belongs to the *Togaviridae* family (genus *Alphavirus*), and was first isolated in 1952–53 from mosquitos and from human serum during an epidemic in Tanzania (Robinson, 1955). CHIKV causes a self-limiting disease known as Chikungunya fever (CHIKF) that is characterized by high fever, rash, myalgia, polyarthralgia and headaches (Burt et al., 2017). While many of the symptoms generally disappear within 1 week, joint pain can persist in some patients for up to a few years (Javelle et al., 2015; Rodriguez-Morales et al., 2016). Over the past decade, the disease caused by CHIKV re-emerged as a serious public health problem and resulted in several outbreaks around the world (Wahid et al., 2017). Although CHIKV has been studied for over 60 years, little is known about immunopathogenesis caused by this virus and about protective immune response against it. In this review, we briefly outline the characteristics of CHIKV, including its structure, transmission, epidemiology, and diagnosis. We then focus on the innate and adaptive immune responses and soluble immunological markers.

## Chikungunya virus

CHIKV is an enveloped alphavirus of ~60–70 nm in diameter. It has an 11.8 kb-long single-stranded positive-sense RNA genome that encodes six structural (C-E3-E2-6K/TF-E1; Metz and Pijlman, 2016) and four non-structural (nsP1, helicase nsP2, nsP3 and polymerase nsP4; Ahola and Merits, 2016) proteins. Genomic RNA associates with 240 copies of 261 amino acid-long structural capsid protein C that forms icosahedral nucleocapsid (Khan et al., 2002; Jose et al., 2009). E1 and E2 are surface glycoproteins, 439 and 423 amino acid-long, respectively (Khan et al., 2002). E1 and E2 carry the major viral epitopes and participate in the attachment and the entry of the virus into target cells, where E2 is responsible for receptor binding, and E1–for membrane fusion (Voss et al., 2010). E3 consists of 64 amino acids that are required for E3-E2-6K-E1 or E3-E2-TF polyprotein translocation into the endoplasmic reticulum for virus spike formation (Snyder and Mukhopadhyay, 2012). The 61 amino acid-long 6K protein is a cation-selective ion channel that is responsible for increased cell permeability to monovalent cations and virion budding during infection (Melton et al., 2002). Transframe protein TF is produced as a result of C-terminal extension of 6K protein in the −1 frame (Firth et al., 2008). It retains ion-channel activity similar to that of 6K and appears to be important for the virus particle assembly and release (Snyder et al., 2013). Although the non-structural proteins nsP1-nsP4 are primarily associated with the viral replication process (Solignat et al., 2009; Lum and Ng, 2015), they carry out additional functions during the viral infection, just like in other alphaviruses (Rupp et al., 2015). It is worth noting that non-structural proteins are not packaged into the final virions, and hence the structural proteins (mainly surface glycoproteins E2 and E1) are the key targets of the host humoral immune response and of most anti-CHIKV vaccines (Powers, 2018).

## Epidemiology and vectors

CHIKV is a zoonotic virus that uses several non-human primates (NHPs) and possibly other vertebrates as amplification hosts (Tsetsarkin et al., 2016), which could also serve as virus reservoirs (Althouse et al., 2018) during inter-epidemic periods.

The first reported case of CHIKV human infection happened in Tanzania in 1952–53. Since then, several outbreaks occurred throughout the African continent (Robinson, 1955; Powers et al., 2000). Between 1960 and 1980, the virus was identified in Central, Western and Southern Africa (Powers et al., 2000), and in following years—in India and other countries of Asia and Africa (Wahid et al., 2017). Phylogenetic reconstruction of CHIKV evolution identified Asian, East/Central/South African (ECSA) and West African lineages which until 2004 (Sam et al., 2015) were mostly confined to the geographic regions after which they were named (Schuffenecker et al., 2006; Sudeep and Parashar, 2008; Wahid et al., 2017).

**Figure 1:**
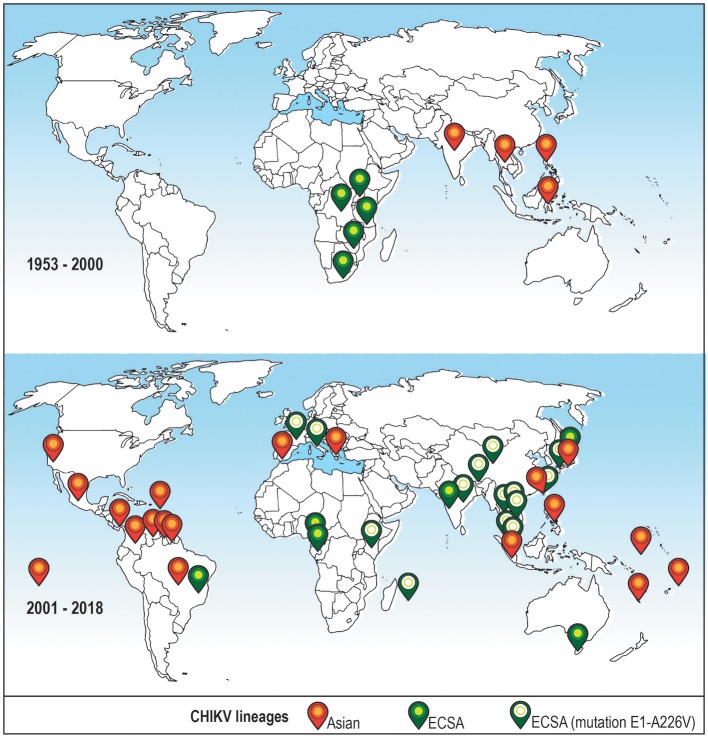
Distribution of CHIKV lineages that are associated with recent outbreaks around the world before and after year 2000. Top: CHIKV cases reported in the XX century (1953–2000). Bottom: CHIKV cases reported recently (2001–2018). Only cases where the virus lineage was identified are shown. Orange icon–Asian lineage; green icon–East/Central/South African (ECSA) lineage; green icon with a white circle–ECSA strain with a mutation A226V in the E1 envelope glycoprotein, this strain is sometimes referred to as Indian Ocean lineage (Wahid et al., 2017).

The first case of autochthonous transmission of CHIKV in the Americas was reported on Saint Martin Island in 2013 (Leparc-Goffart et al., 2014), and it was shown that the risk exists that CHIKV will establish enzootic/sylvatic cycle in the tropical regions of the American continent (Lourenço-de-Oliveira and Failloux, 2017). Increased traveling in the recent years and the presence of appropriate vectors allowed for a further spread of CHIKV with reports from the United States (Kendrick et al., 2014), Brazil (Tanabe et al., 2018), Spain (Bocanegra et al., 2016), Italy (Zammarchi et al., 2016) and Australia (Huang et al., 2017), among others (Wahid et al., 2017). CHIKV virus spread and outbreaks around the world in the last years are shown on Figure 1.

CHIKV disease (CHIKVD) has enzootic/sylvatic and urban cycles of transmission (Weaver, 2013) and occurs through a bite of infected female mosquitoes of *Aedes* genus (Sudeep and Parashar, 2008). *Aedes aegypti* and *Aedes albopictus* are the two most significant and well-documented CHIKV vectors, associated with outbreaks worldwide (Mourya and Mishra, 2006). The urban cycle of transmission is possible because of the sufficiently high levels of viremia developed in the infected individuals (Go et al., 2014) and it can start with the spillover of enzootic/sylvatic CHIKV via bridge vectors, such as *Aedes furcifer* (Diallo et al., 2012). The spread of CHIKV in the United States and Europe was linked to the adaptation of the ECSA strains to *Aedes albopictus* mosquitoes that are abundant in these regions (Madariaga et al., 2016). This adaptation to a different vector was attainable due to a mutation in the envelope protein gene (E1-A226V; Tsetsarkin et al., 2007, 2011), which is sometimes regarded as giving rise to Indian Ocean lineage (Wahid et al., 2017). Several other mutations that further enhance fitness and adaptation of CHIKV to its hosts were identified in E1 and E2 proteins (Singh et al., 2012; Agarwal et al., 2016), and were shown to occur in the intrinsically disordered regions of these proteins (Singh et al., 2018).

Cases of maternal-fetal transmission were reported (Ramful et al., 2007; Gérardin et al., 2008; Economopoulou et al., 2009) and the virus was detected in human breast milk (Campos et al., 2017), although the data on the impact of the infection is somewhat controversial (Laoprasopwattana et al., 2015; Torres et al., 2016), and experimental data from Rhesus macaques (*Macaca mulatta*) speaks against possibility of trans-placental transmission (Chen et al., 2010).

## Diagnosis of CHIKV infection

**Figure 2:**
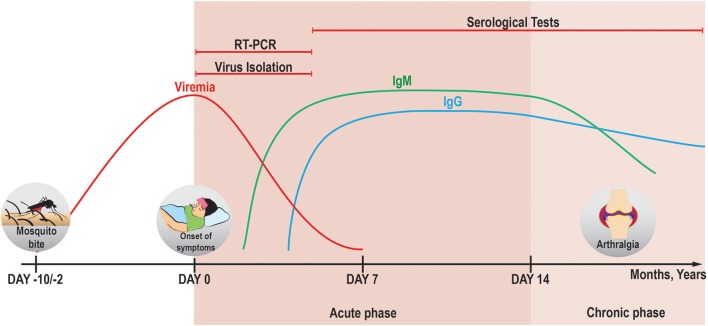
Applicability of different diagnostic methods in the course of CHIKV infection. In the acute phase, viremia can persist until days 5–7 *pio* (Silva and Dermody, 2017) and CHIKV genomic RNA can be detected by RT-PCR reliably until day 7 *pio* (Edwards et al., 2017). It is therefore suggested that the detection of CHIKV RNA and virus isolation from serum samples for diagnostic purposes is done before day 5 *pio* (Johnson et al., 2016b), because the chance of false-negative results increases with the decrease in viral load. IgM and IgG antibodies against CHIKV begin to be produced at days 2 *pio* (Jain et al., 2018) and 4 *pio* (Prince et al., 2015), respectively. Stable titers of IgM can be seen in the serum from day 6 *pio* till around 4 months *pio* (Prince et al., 2015) [and can be detected mostly until 6 months *pio* (Chua et al., 2017)], whereas sustained levels of IgG can be present for more than 1 year (Chua et al., 2017). The antibodies against CHIKV can be detected by immunoassays after the development of humoral immune response (in case of IgG–long into the chronic phase, both–symptomatic or asymptomatic). A more detailed overview of the methods available for diagnostics of CHIKV is given in a review by Sam et al. (2015).

To date, several different methods are used to diagnose the CHIKV infection. These methods are based on the detection of (i) viral RNA (Pfeffer et al., 2002; Pastorino et al., 2005), (ii) IgM and IgG antibodies against the virus (or viral antigens proper; Kashyap et al., 2010; Johnson et al., 2016a,b), or (iii) viral particles in the conditioned media of cell lines that were exposed to samples of patients' serum *in vitro* (Pan American Health Organization, 2011). It is important to keep in mind that the detection efficiency of these methods varies depending on both the presence of the viral particles in the bloodstream of a patient and on the time of sample collection (Figure 2).

## Pathology of CHIKV infection

The incubation period of 2–10 days is usually followed by CHIKVD that can be divided into acute and chronic phases. The acute phase occurs during the first 2 weeks after the onset of the disease and can be further subdivided into viral (before day 5 *p*ost-*i*llness *o*nset, *pio*) and convalescent (days 5–14 *pio*) stages (Thiberville et al., 2013a). Polyarthralgia, the most characteristic symptom of the acute phase, is reported in 87–98% of cases (Thiberville et al., 2013b). When and if the disease continues into the extended symptomatic-chronic phase, arthralgia that usually affects multiple joints can remain for several months or even years (Moro et al., 2012; Schilte et al., 2013).

**Figure 3:**
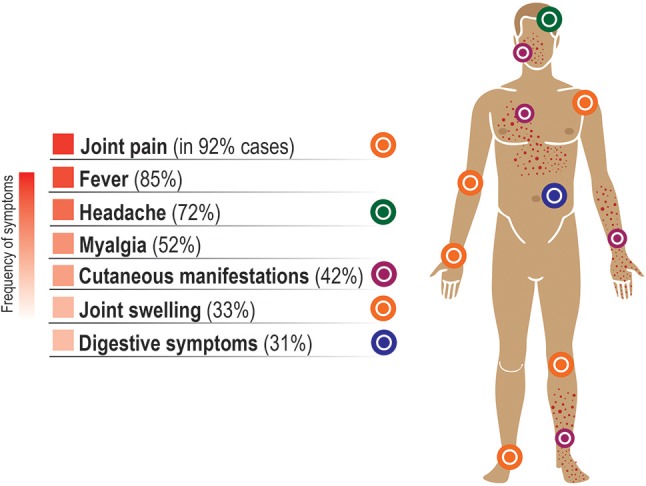
Symptoms of the acute phase of CHIKV infection. Some minor variation exists in the frequency of symptoms reported in different studies. Typically, the clinical symptoms in the acute phase of the disease include high fever, pain and swelling in the joints, myalgia, and skin rash, often accompanied by headache, backache and fatigue. Here, the average percentage of symptomatic cases where a given symptom was reported is based on the data from Thiberville et al. (2013b), with the exception for the percentages for fever and headache that were taken from Huits et al. (2018). These symptoms usually remain for about 5–7 days as a self-limiting disease and are followed by a complete recovery within 2 weeks. However, severe joint pain can remain for months or even years in some individuals, often in distal joints (Roosenhoff et al., 2016) and in fluctuating manner (Hoarau et al., 2010). It has been estimated that ~16% of cases are asymptomatic (Thiberville et al., 2013b). The symptoms in CHIKV-infected children differ from those in adults and are listed in Table 1 from the study by Simarmata et al. (2016).

Generally, acute clinical symptoms include high fever (>38.5°C) and shivers, severe joint and muscle pain, skin rash, weakness and headache (Figure 3). High viral load, lymphopenia and moderate thrombocytopenia are also observed in the acute phase (Thiberville et al., 2013b). In most cases, the symptoms remain for about 4–7 days as a self-limiting disease and are followed by a complete patient recovery (Schwartz and Albert, 2010). Nonetheless, clinical cases of symptomatic chronic disease for up to several years were reported (Brighton et al., 1983; Borgherini et al., 2008; Soumahoro et al., 2009; Gérardin et al., 2011; Moro et al., 2012; Schilte et al., 2013). Studies conducted after CHIKV outbreaks on Reunion Island in 2006 and in Italy in 2007 showed persistence of myalgia, asthenia and arthralgia in 60–67% of cases 36 and 12 months post-infection, respectively (Moro et al., 2012; Schilte et al., 2013).

Newborns and infants are predisposed to develop a more severe disease (Sebastian et al., 2009; Thiberville et al., 2013b). Fever is the main symptom in children (Simarmata et al., 2016). Both atypical and severe cases were frequently observed in this patient group, with the disease leading to hyperpigmentation, erythema (Nair, 2008; Rao et al., 2008), bullous skin lesions (Robin et al., 2010) and neurological symptoms, such as seizures and encephalitis (Robin et al., 2008), and a variety of other complications (Ramful et al., 2007).

Elderly individuals are another group with an increased risk of a more severe progression of the CHIKVD. Recently reported fatal cases of CHIKV infection in elderly people described liver failure with subsequent cardiovascular collapse (Chua et al., 2010), and neurological and pulmonary deterioration followed by multiple organ failure (Hoz et al., 2015). Furthermore, 65 fatal cases after atypical CHIKV infection were reported during the 2006 outbreak on Reunion Island (Economopoulou et al., 2009). Mortality rate and overall severity of the disease increased there with age (Josseran et al., 2006), which seems to be a common theme for CHIKV infection in humans (Hoarau et al., 2010; Lang et al., 2017) and NHPs (Messaoudi et al., 2013). The incidence of atypical cases of CHIKF reported previously was <1%, taking into consideration the total number of infected people (Economopoulou et al., 2009). Some of the major atypical cases of CHIKV infection (as of 2010) are summarized in the review by Rajapakse et al. (2010).

After the mosquito bite, CHIKV replicates at the site of the inoculation and then spreads to peripheral organs and target cells via the circulatory system (Mourya and Mishra, 2006). CHIKV is able to infect a variety of adherent model cell lines and primary cells, but it fails to either infect or even bind to both–blood-derived cell lines (Jurkat, THP-1, U937, B-420) and primary blood cells (lymphocytes and monocyte-derived dendritic cells) (Sourisseau et al., 2007). Conflicting data is published by Sourisseau et al. (2007) and by Her et al. (2010) regarding susceptibility of primary human monocytes to CHIKV. Notably, monocyte-derived macrophages were found to be susceptible to CHIKV infection (Sourisseau et al., 2007; Solignat et al., 2009).

Similar to its behavior in humans, in a mouse model, CHIKV has a pronounced tropism to fibroblasts of the muscle, joint connective tissue and deep dermis (Couderc et al., 2008). From the infected skin fibroblasts and dermal macrophages, the virus spreads to lymph nodes, reaching spleen and liver in the acute phase, and muscles and joints through blood—later in the course of the disease (Roosenhoff et al., 2016). In severe cases—the spread of the virus into the central nervous system was shown in a mouse model (Couderc et al., 2008; Gardner et al., 2010) and in cynomolgus macaques (*Macaca fascicularis*) (Labadie et al., 2010).

## Immune response to CHIKV

Innate immune response against viruses consists of macrophages, dendritic cells (DCs) and natural killer cells (NKs) and is followed by the activation of B and T lymphocyte-mediated adaptive immune response. The subsequent generation of memory cells then leads to a specific response to the viral infection and protects from reinfection. In the following sections we review the current knowledge of cellular and molecular immune responses to CHIKV in humans and animal models.

### Innate immune response

#### Natural killer (NK) cells

Acute phase of CHIKVD is marked by a significant increase in activation of components of the cell-mediated immunity led by an extensive activation of innate NK cells (Hoarau et al., 2010). The function of NK cells is regulated by a combination of signals from activating (e.g., CD94/NKG2C and NKG2D, activating killer cell immunoglobulin-like receptors KIRs–KIR2DS, KIR3DS) and inhibitory receptors (e.g., CD94/NKG2A, inhibitory KIRs–KIR2DL, KIR3DL) on their cell membranes (Pegram et al., 2011).

Recent data associate the expression of KIR and NKG2 receptors with susceptibility (Petitdemange et al., 2014) to CHIKV infection and viral clearance (Petitdemange et al., 2011). KIRs are involved in recognition of human leukocyte antigen (HLA) class I molecules (HLA-A, -B and -C) on nucleated cells, and specific KIR ligand/receptor combinations were implicated in HIV and hepatitis C (Jamil and Khakoo, 2011). In a similar fashion, a significant increase in the frequency of HLA-C subtype 2 allele (HLA-C2) in combination with the expression of *KIR2DL1* gene (encodes the receptor that can interact with HLA-C2) was found in CHIKV-infected patients during the CHIKV outbreak in Gabon in 2010 (Petitdemange et al., 2014).

At the same time, high viral load during the acute phase of infection and subsequent clearance of the infected cells were both associated with the expansion of the subpopulation of CD3^−^CD56^+^ NK cells that co-expressed the activating NKG2C receptor and KIR2DL2/KIR2DL3 inhibitory receptors for HLA-C subtype 1. This NKG2C^+^ subpopulation of NK cells rapidly increased in the acute phase (at the expense of NKG2A^+^ population) and demonstrated strong cytolytic response and reduction in IFN-γ production. This argues for a dichotomy between cytolytic and immunoregulatory functions of NK cells in the acute phase of infection (Petitdemange et al., 2011). In contrast, compared to controls, NK and NKT-like (CD3^+^CD56^+^) cells had lower cytotoxicity and higher expression of IFN-γ in the chronic phase. In addition, more of these cells expressed the inhibitory NKG2A receptor, while fewer were positive for the activating NKG2D (Thanapati et al., 2017).

Strong cytolytic response and decreased responsiveness to cytokine stimulation are typical for terminally differentiated NK cells that mature in progression from CD56^bright^CD57^−^ to CD56^dim^CD57^−^ and then—to CD56^dim^CD57^+^ phenotype (Nielsen et al., 2013). In agreement with that, the shift from CD56^bright^ to cytolytic and mostly unresponsive to cytokines CD56^dim^ cells was observed among CD3^−^CD56^+^ NK cells in CHIKV-infected patients (Petitdemange et al., 2011). The number of terminally differentiated CD57^+^ NK cells peaked at the early (up to day 3 *pio*) acute phase. Persistence of these NK cells correlated with the viral load, and extended past the day 30 *pio* in some patients, all of which later developed chronic arthralgia (Petitdemange et al., 2016). Interestingly, acute joint pathology in CHIKV-infected mice was associated with NK cell activity that also becomes detectable in the early acute phase of the disease (Teo et al., 2015).

#### Monocytes, macrophages and dendritic cells (DCs)

Monocytes and monocyte-derived macrophages appear to play a central role in the CHIKV-associated joint pathology. During CHIKV outbreaks, patients generally develop polyarthritis as an arthritis-like syndrome (Amdekar et al., 2017) in synovial joints (Phuklia et al., 2013). The inner lining of synovial joints is formed by macrophage-like synovial cells and fibroblast-like synoviocytes, and the latter are known to be important for the pathogenesis of rheumatoid arthritis (Bartok and Firestein, 2010). Similar to primary human osteoblasts (Noret et al., 2012), *in vitro* cultures of primary human fibroblast-like synoviocytes are susceptible to CHIKV infection, which results in the secretion of IL-6, IL-8, CCL2/MCP-1 and RANKL by the infected cells. The supernatants from these CHIKV-infected synoviocytes induce migration of monocytes as well as differentiation of monocytes/macrophages into osteoclast-like cells that produce high levels of arthritis mediators, such as IL-6 and TNF-α (Phuklia et al., 2013). In the joint, osteoclast-like cells can damage joint structure and contribute to the arthritic-like syndrome, as it was shown for rheumatoid arthritis (Schett, 2007).

In the chronic phase, macrophages were proposed to act as cellular reservoirs of persistent CHIKV (Labadie et al., 2010) and as regulators of the local Th1/Th2 balance (Dupuis-Maguiraga et al., 2012) in the damaged tissues that they infiltrate together with other mononuclear inflammatory cells. Such infiltrates were observed in the muscles of CHIKV-infected mice (Ziegler et al., 2008), in the muscles, joints, lymphoid tissues and liver of the infected macaques (Labadie et al., 2010), and in human biopsy samples. For example, in one chronic patient, both CHIKV RNA and proteins in the perivascular synovial macrophages were detected 18 months post-infection. While the synovial fluid contained activated CD56^+^ NK and CD4^+^ T cells, the majority (~50%) of infiltrating cells were CD14^+^ monocytes (Hoarau et al., 2010). Virus persistence and active monocyte trafficking into the synovial tissue and fluid were associated with the robust expression of IFN-α (Hoarau et al., 2010), a potent inhibitor of CHIKV replication (Sourisseau et al., 2007). In agreement with that, high levels of IFN-α were produced by monocytes and whole blood cultures that were infected with CHIKV *in vitro* (Her et al., 2010). The importance of monocytes for limiting CHIKV infection is further illustrated in a mouse model, where depletion of Ly6C^hi^ CCR2^+^ (receptor for CCL2/MCP-1) monocytes *in vivo* promoted a more severe disease (Haist et al., 2017).

In a mouse model, macrophages were shown to be important for both–viral clearance and development of arthritic symptoms (Gardner et al., 2010). Similar to human primary macrophages (Sourisseau et al., 2007), primary mouse macrophages (Gardner et al., 2010) and transformed RAW264.7 mouse macrophage cells (Kumar et al., 2012b; Nayak et al., 2017) are also susceptible to CHIKV *in vitro*. Nonetheless, in the two recent studies conflicting results were obtained regarding CHIKV ability to cause apoptosis in the infected cells. No apoptosis was observed by Kumar et al. (2012b), while the study by Nayak et al. reported apoptosis induction through both–intrinsic and extrinsic pathways (Nayak et al., 2017). Both studies recorded an upregulation of IL-6 and TNF-α levels (Kumar et al., 2012b; Nayak et al., 2017). We note here, that two protective mechanisms of the host—apoptosis and autophagy—can play both pro- and antiviral roles in CHIKV infection, as discussed in the review by Long and Heise (2015).

DCs, another monocyte-derived cell type, participate in antigen presentation and therefore connect the innate and adaptive immune responses. Although shown to be susceptible to CHIKV in cynomolgus macaques (Labadie et al., 2010), cultures of primary human DCs (unlike monocyte-derived macrophages) appear to be resistant to CHIKV (Sourisseau et al., 2007). There are only a few studies related to the interaction of DCs with CHIKV. One of those assessed the role of dendritic cell immunoreceptor (DCIR) in CHIKV infection in mice. In this work, CHIKV decreased the number of DCIR^+^ cells at the site of infection. It also altered cytokine expression in cultures of bone marrow-derived dendritic cells from DCIR^−/−^ mice. In addition, infected DCIR^−/−^ mice developed more severe disease symptoms, such as edema, increased inflammation and weight loss, suggesting a role for this receptor in limiting CHIKV-induced inflammatory response (Long et al., 2013). In another study, intracerebroventricular injection of CHIKV in neonate mice promoted the infection of astrocytes that was accompanied by a robust mobilization of DCs restricted to the site of infection (Das et al., 2015).

It is important to mention, that the current understanding of the cell-mediated immune responses to CHIKV is often based on the research done in animal models [reviewed comprehensively elsewhere (Broeckel et al., 2015; Fox and Diamond, 2016; Haese et al., 2016)], and none of those models completely recapitulates the course of CHIKVD in humans (Roosenhoff et al., 2016). In line with that, the roles of the less studied immune cell types, such as γδ T cells (Long et al., 2016), neutrophils and eosinophils (Poo et al., 2014) to our knowledge are only described in the context of CHIKVD in mouse models. Therefore, further research is required to fully understand the role of those cell types in the protection from or development of CHIKV-associated pathology in humans.

### Humoral and cellular adaptive immune response to CHIKV

Pathogen-specific, humoral and cell-mediated immune responses that together constitute the adaptive immunity are carried out by B and T lymphocytes, respectively. An induction of anti-CHIKV antibodies that subsequently led to rapid clearance of the virus was demonstrated in a mouse model. In accordance with that, B cell (μMT) knockout mice showed a more severe disease and persistent viremia (for over a year) highlighting the importance of these antibody-producing cells for CHIKV clearance (Lum et al., 2013). In Rag1^−/−^ mice that lack both B and T cells, prophylactic administration of anti-CHIKV monoclonal antibodies was sufficient to prevent virus persistence (Hawman et al., 2013). In addition, therapeutic administration of a human neutralizing monoclonal antibody in rhesus monkeys at days 1 and 3 after CHIKV infection blocked virus spread and inflammation in several tissues including joints and muscles (Broeckel et al., 2017).

In humans, anti-CHIKV IgG is first detected in the early convalescent stage, when naturally-acquired IgG response is dominated by the antibodies of IgG3 subtype. Early appearance of these antibodies correlates with protection against complications of the chronic CHIKVD (Kam et al., 2012b).

Both in humans and mice the antibody-mediated immune response seems to primarily target the envelope glycoprotein E2 of CHIKV (Kam et al., 2012a; Smith et al., 2015; Weber et al., 2015; Weger-Lucarelli et al., 2015). Moreover, the majority (70–80%) of the antibodies are estimated to be directed against the single linear epitope (E2EP3) in the N-terminus of the viral E2 protein. Accordingly, CHIKV infection in mice vaccinated with E2EP3 peptides was characterized with reduced infectivity of the virus and better clinical outcomes with decreases in viremia and joint inflammation. In plasma samples from patients in convalescent and recovery stages, anti-E2 antibodies were also shown to be the most persistent—they were detectable 21 months *pio*, unlike anti-E3, anti-capsid and anti-nsP3 antibodies that had been present only earlier in the course of the disease (Kam et al., 2012a). According to epitope mapping, monoclonal antibodies produced only against the epitopes on the outer surfaces (and not facing the interior of the E2/E1 trimer structure) were neutralizing (Fong et al., 2014).

The role of specific anti-CHIKV antibodies in the disease immunopathology has also been studied. Recently, two peptides of CHIKV E1 glycoprotein were identified by *in silico* bioinformatic analysis and showed similarity to human proteins. These E1 peptides were recognized by the serum from CHIKV-infected patients and were able to induce muscle inflammation in mice, thus showing that molecular mimicry between virus and host proteins contributes to CHIKV pathology (Reddy et al., 2017). In another study, sub-neutralizing levels of CHIKV-specific antibodies aggravated the disease in mice, showing thereby that antibody-mediated enhancement of CHIKVD severity is also possible and requires consideration (Lum et al., 2018).

Cytotoxic CD8^+^ T cells represent one of the major resources of antiviral immunity and are responsible for destruction of the infected cells. Analysis of circulating T lymphocytes showed that in acutely infected patients the early stage of the CHIKVD is accompanied by activation and proliferation of CD8^+^ T lymphocytes with a peak at day 1 *pio* (Wauquier et al., 2011). Higher percentages of activated CD8^+^ cells remained in the blood 7–10 weeks post-infection in the patients with CHIKV-associated arthritis symptoms. Elevated numbers of CD8^+^ cells, as compared to healthy controls, were also observed in patients with untreated rheumatoid arthritis (Miner et al., 2015).

While CD8^+^ T cell response marks the early stage of CHIKV infection, CD4^+^ T cell lymphocyte-mediated immune response increases toward the end of the acute phase, peaking at day 4 *pio* (Wauquier et al., 2011).

The main function of CD4^+^ T helper cells is to support and modulate the activity of other immune cells. This is achieved via production of cytokines that stimulate cell-mediated immunity and antibody responses. The role of these cells in CHIKV infection was studied in CD4^−/−^ and CD8^−/−^ KO mice. CHIKV-specific CD4^+^ and not CD8^+^ T cells were directly linked to the IFN-γ-independent inflammation in the joints, without evident role in replication and dissemination of the virus in the body (Teo et al., 2013). Moreover, a transfer of splenic CD4^+^ T cells from CHIKV-infected wild-type mice into T cell receptor-deficient CHIKV-infected mice promoted a severe joint disease in the latter, further illustrating the essential role of CD4^+^ T cells in the CHIKV-associated joint inflammation (Teo et al., 2017).

Therapeutic strategies based on the inhibition of CD4^+^ T cells were developed and proved to be promising. For example, a treatment with FTY720 (fingolimod), an agonist of a phosphorylated sphingosine 1-phosphate receptor 1 (S1PR1), successfully abrogated joint pathology in CHIKV-infected mice by blocking the S1PR1-mediated emigration of CD4^+^ T cells from the lymph nodes into the joints (Teo et al., 2017). In a parallel study, CHIKV-infected mice were treated with a fusion protein CTLA4-Ig (abatacept) that blocks costimulatory receptors on the surface of T cells and prevents activation of the latter. The treatment resulted in decreased inflammation and lower numbers of CD11b^+^/Ly6C^+^ monocytes, NK and T cells in the joints of the infected animals. Although unsuccessful at completely clearing the viral RNA, a combined therapy with CTLA4-Ig and anti-CHIKV monoclonal antibodies quickly eliminated the infectious virus and further improved disease pathology (Miner et al., 2017). Both works focused on acute joint pathology and called for testing of abovementioned approaches for the treatment of the chronic CHIKV-induced arthritis (Miner et al., 2017; Teo et al., 2017).

## Cytokines as immunological markers in CHIKV-associated disease

**Figure 4:**
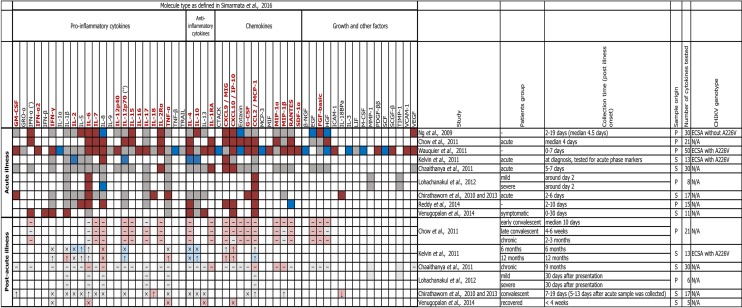
Profile of pro- and anti-inflammatory cytokines, chemokines and growth factors in human CHIKV infection. The heatmap shows soluble immune mediators that were measured in patients during the acute and post-acute phases (where available) of CHIKVD in ten studies. For comparisons between CHIKV-infected and healthy individuals, dark and light colors are used to highlight the differences or lack of thereof during the acute and post-acute phases, correspondingly; red indicates significant upregulation, blue–significant downregulation, gray–lack of significant differences in the levels of a given cytokine/chemokine/growth factor, and white indicates that those levels were not measured. For comparisons between acute and post-acute phases, ↓ and ↑ symbols indicate down- and upregulation in the post-acute phase, × symbol indicates the absence of significant differences, and “–” (en dash) symbol indicates that no comparison was reported. Where not specified, we assumed that: IL-12 is IL12p70 ('), and IFN-α is IFN-α1 (''). Patients group names are given as in the corresponding study. Sample origin: S–serum, P–plasma. CHIKV genotype: ECSA–East/Central/South African, N/A–no data. Names of the factors that are shown to be age-independently upregulated in the acute phase of CHIKVD by Simarmata et al. (2016) are shown in dark red. References: (Ng et al., 2009; Chirathaworn et al., 2010, 2013; Chaaitanya et al., 2011; Chow et al., 2011; Kelvin et al., 2011; Wauquier et al., 2011; Lohachanakul et al., 2012; Reddy et al., 2014; Venugopalan et al., 2014).

A vast number of samples were collected during the recent outbreaks around the world. In combination with increasing availability of high-throughput screening platforms, this allowed researchers to link various aspects of CHIKVD to expression profiles of cytokines, chemokines and growth factors in humans. We outline these expression profiles on Figure 4 and summarize the principal findings below.

Ng et al., Wauquier et al., and Reddy et al. used plasma samples from acutely infected patients and uninfected individuals to compare levels of cytokines, chemokines and growth factors. The study by Ng et al. analyzed the levels of 30 such molecules, of which 12 were upregulated, and 4–downregulated (Ng et al., 2009). A similar comparison by Wauquier et al. included 50 soluble proteins, of which 25 were upregulated (including ICAM-1, VCAM-1, and RANTES that were undetectable in controls) and 10–downregulated. Notably, the exclusion of older individuals from the analysis did not affect these results. Many of the upregulated proteins (e.g., IFN-γ, IL-6, CXCL10/IP-10, CCL2/MCP-1, and others) showed dynamic expression pattern with levels changing across the sampling timeline (day 0–7 *pio*). The levels of many cytokines varied sufficiently not only at different time points, but also between individuals. IFN-α2, whose inter-individual levels were somewhat homogenous, represented one of the few exceptions (Wauquier et al., 2011). Out of 15 cytokines and chemokines tested by Reddy et al. (2014), seven were upregulated, one was downregulated, and only the upregulation of IL-6 and CXCL10/IP-10 was in agreement with both of the abovementioned studies (Ng et al., 2009; Wauquier et al., 2011).

Dynamic expression of cytokines and chemokines is not exclusive to the early acute phase. Venugopalan et al. showed that Th1 cytokines (e.g., IFNs -α, -β, -γ, IL-1β, CXCL10/IP-10, CCL2/MCP-1) reach maximum levels between days 0 and 5 *pio*, and Th2 cytokines (e.g., IL-4, IL-13)—between days 15 and 30 *pio* (Venugopalan et al., 2014). Chow et al. also reported the bias toward Th2 cytokines in the early convalescent stage (around 10 days *pio*). In the same study, the levels of RANTES and EGF peaked in the late convalescent stage (4–6 weeks *pio*) and of IL-17—in the chronic phase (2–3 months *pio*) (Chow et al., 2011). Later in the chronic phase Kelvin et al. found that high IgG levels were accompanied by increased levels of IL-6, CXCL9/MIG, and CXCL10/IP-10 in the 6-months follow-up of CHIKVD patients. In the 12-months follow-up, high IgG levels coincided with higher levels of CXCL9/MIG and lower—of IL-10 (Kelvin et al., 2011).

The relationship between the expression profile of cytokines and chemokines in the early phases of CHIKVD and the severity of the disease was studied in various contexts and yielded different results. The severity of symptoms in the 2007 outbreak in Singapore was associated with high levels of IL-1β and IL-6 and a decrease in the level of RANTES (Ng et al., 2009). At the same time, in the 2007 outbreak in Italy the severity of CHIKVD was associated with increased levels of CXCL9/MIG, CXCL10/IP-10, and IgG (Kelvin et al., 2011). During the 2009–2010 outbreak in Thailand, the severity of CHIKVD was linked to elevated levels of IL-6 and CCL2/MCP-1 and decreased levels of IL-8 (Lohachanakul et al., 2012). In CHIKV-infected mice, the expression levels of monocyte chemoattractant proteins MCP-1/CCL2, MCP-2/CCL8, and MCP-3/CCL7 were increased in joints and the treatment with MCP inhibitor reduced the virus-induced bone loss in these animals (Chen et al., 2015).

Other relationships of cytokine and chemokine levels in the course of CHIKVD were established as well. For example, strong Th2 cytokine response was associated with prolonged presence of musculoskeletal symptoms of CHIKVD (Venugopalan et al., 2014), and increased levels of IL-6 (Chaaitanya et al., 2011; Chow et al., 2011) and of GM-CSF (Chow et al., 2011)—specifically with persistent arthralgia.

Another interesting observation regards the correlation of cytokine and chemokine levels with viral loads. High viral load positively correlates with the levels of IFN-α, IL-6, IL-12, IL-1RA, CXCL10/IP-10, and CCL2/MCP-1 (Chow et al., 2011), which seems to be in agreement with the recent data from Teng et al. (2015). Similarly, CCL2/MCP-1 correlated strongly, and IL-6—moderately with high viral load in the patients in the acute phase that were positive for CHIKV RNA (Reddy et al., 2014).

A comprehensive catalog of genes that are differentially expressed upon CHIKV infection was obtained by using RNAseq in a mouse model. Gene expression changes were in agreement with previously published mouse, monkey and human studies, and allowed for identification of an emerging role for granzyme A in CHIKV-associated arthritis (Wilson et al., 2017). Nonetheless, we would like to emphasize that extreme caution should be taken when extrapolating conclusions from studies in animal models to humans. For example, anti-CHIKV IgG3 antibodies, abundant in humans (Kam et al., 2012b), were not detected in mice at all (Patil et al., 2012; Teo et al., 2015). Opposite to humans (Venugopalan et al., 2014), the cytokine response in mice is shifted from Th2 early in the acute phase (Patil et al., 2012; Teo et al., 2015) toward Th1 later in the course of the disease (Patil et al., 2012). This early Th2 response was further enhanced when mice were infected via mosquito bite (Thangamani et al., 2010; Saraswat et al., 2016). This finding highlights the importance of both–the choice of the animal model and of the virus transmission route in the experimental system.

The authors of the abovementioned works further discussed the roles of Th1 and Th2 cytokine responses (Venugopalan et al., 2014), type I and II IFN signaling (Chirathaworn et al., 2010; Wauquier et al., 2011; Long and Heise, 2015), and the involvement of individual cytokines and chemokines: IL-6 (Chow et al., 2011; Kelvin et al., 2011), IL-13 (Venugopalan et al., 2014), IL-7, IL-15, RANTES (Ng et al., 2009), IL-18 (Chirathaworn et al., 2010), IL-1β, TNF-α, CXCL9/MIG, CXCL10/IP-10, and CCL2/MCP-1 (Kelvin et al., 2011). Simarmata et al. elaborated on the association of lower levels of pro-inflammatory cytokine GM-CSF with joint pain in CHIKV-infected children (Simarmata et al., 2016). Recent work by Chen et al. also connected the activation of NLRP3 inflammasome by CHIKV with upregulation of IL-1β and IL-18, as well as with the inflammation and osteoclastogenic bone loss in the CHIKVD (Chen et al., 2017).

Several explanations were suggested for discrepancies between cytokine, chemokine and growth factor expression profiles observed in patients with CHIKVD. One variable that needs to be considered is the genotype of the virus, which can affect the degree of joint pathology, the extent of inflammatory cells infiltration, and the intensity of cytokine response (Teo et al., 2015). Other important factors were pointed out by the authors of the studies that are shown here on Figure 4. In particular, attention was drawn to the differences in experimental approaches (Wauquier et al., 2011; Venugopalan et al., 2014), cohort sizes (Chow et al., 2011; Wauquier et al., 2011; Venugopalan et al., 2014), genetic backgrounds (Kelvin et al., 2011; Reddy et al., 2014), disease stages included into analyses (Chirathaworn et al., 2013), disease severity (Reddy et al., 2014), sources (e.g., plasma or serum) of the samples (Teng et al., 2015) and their collection times (Chirathaworn et al., 2013; Venugopalan et al., 2014).

## CHIKV vaccines and anti-CHIKV monoclonal antibodies

As of September of 2018, after over 50 years of development, there are no licensed vaccines or antiviral therapeutic strategies for prevention or treatment of CHIKV infection (Ljungberg et al., 2016; Powers, 2018).

The first formalin-inactivated CHIKV vaccine was produced in the culture of green monkey kidney tissue in the 1970s and was shown to be tolerated and immunogenic in 16 healthy human adults (Harrison et al., 1971). Recently, a formalin-inactivated CHIKV vaccine produced in Vero cells neutralized the virus infectivity by stimulation of both humoral and cellular immune response in the immunized mice (Tiwari et al., 2009). Additionally, both recombinant E2 protein and whole-inactivated virus vaccines protected mice from CHIKV infection, and no virus was detected in the tissues of immunized animals (Kumar et al., 2012a).

Over the years, several vaccine candidates were evaluated as possible preventive approaches. Among those are inactivated (Rudd et al., 2015; DeZure et al., 2016) and live attenuated (Edelman et al., 2000; Plante et al., 2011; Chu et al., 2013; Hallengärd et al., 2014; Roy et al., 2014; Roques et al., 2017) viruses, DNA (Mallilankaraman et al., 2011; Bao et al., 2013; Hallengärd et al., 2014; Tretyakova et al., 2014; Muthumani et al., 2016; Roques et al., 2017) and subunit (Metz et al., 2011, 2013; Khan et al., 2012) vaccines, as well as vaccines that are based on virus-like particles (VLPs) obtained from yeast (Saraswat et al., 2016), insect (Metz et al., 2013) and mammalian cells (Akahata et al., 2010; Chang et al., 2014). A large group of promising vaccine candidates takes advantage of chimeric avirulent backbones of measles (Brandler et al., 2013; Ramsauer et al., 2015) and vaccinia viruses (García-Arriaza et al., 2014; Weger-Lucarelli et al., 2014), adenovirus (Wang et al., 2011a), vesiculovirus (Chattopadhyay et al., 2013) and alternative alphaviruses (Wang et al., 2011b; Erasmus et al., 2017). Candidate vaccines are tested in mouse and NHP models, and some of them have completed—NCT02861586 (Edelman et al., 2000) or are currently in the phase 2 clinical trials NCT02562482 (Chang et al., 2014).

Each of these approaches has its own advantages and limitations, and their immunogenicity should be carefully balanced with tolerability, lack of adverse effects and overall safety. For example, one of the early candidate vaccines was based on the live attenuated CHIKV strain 181/clone 25. In phase 2 clinical trials, it elicited neutralizing antibodies in 98% of recipients by day 28, and 85% of vaccinated individuals remained seropositive 1 year after immunization. Nonetheless, 8% of vaccinees developed transient arthralgia, although without arthritic signs or flu-like syndromes (Edelman et al., 2000).

Recently, vaccine candidates were developed that employ a picornavirus internal ribosome entry site (IRES) to render them incapable of infecting mosquitos and to reduce the expression of CHIKV structural protein genes (Plante et al., 2011; Roy et al., 2014). A single dose of such live attenuated virus was shown to be highly immunogenic and prevented the development of the hyperthermia and acute viremia in cynomolgus macaques (Roy et al., 2014). Interestingly, this Indian Ocean lineage-based vaccine can provide both–protection against other CHIKV lineages (Langsjoen et al., 2018) and cross-species protection against O'nyong'nyong virus (Partidos et al., 2012).

Current antiviral development strategies rely on exploitation of known antiviral agents and chemicals against other pathogens and synthesis of novel compounds (Ravichandran and Manian, 2008; Abdelnabi et al., 2015; Powers, 2018; Subudhi et al., 2018), nucleic acid-based therapies (Dash et al., 2008; Parashar et al., 2013; Lam et al., 2015) and anti-CHIKV monoclonal antibodies (Pal et al., 2013, 2014; Selvarajah et al., 2013; Clayton, 2016; Broeckel et al., 2017). In the last years, the use of monoclonal antibodies (mAbs) as therapeutic agents against CHIKV infection was evaluated by several groups, as reviewed by April M. Clayton (Clayton, 2016). Human anti-CHIKV mAbs were shown to have both–prophylactic and therapeutic effects in an adult wild-type mouse model of CHIKVD (administered 8 or 18 h post-virus challenge; Selvarajah et al., 2013), and to protect immunocompromised *Ifnar1*^−/−^ mice from lethal virus challenge (Smith et al., 2015). Similarly, in the screening of 230 mouse anti-CHIKV mAbs, 36 were found to be neutralizing, of which a combination of two was the most potent in protecting *Ifnar1*^−/−^ mice against CHIKV-induced death (Pal et al., 2013). This combination was used in the follow-up study, where it neutralized infectious CHIKV in blood and reduced viral burden in the joints and muscles of the legs of infected rhesus macaques (Pal et al., 2014). Recently, it was also shown that the treatment of rhesus macaques with an anti-CHIKV mAb (SVIR0001) administered after virus infection reduced viremia, joint disease, cellular inflammatory infiltration and the levels of pro-inflammatory cytokines and chemokines (Broeckel et al., 2017).

## Conclusions

In the light of recent outbreaks, interest in CHIKV from the scientific community has grown significantly. Despite an outstanding progress in the CHIKV research, several questions regarding its immunopathology and associated arthritic syndrome remain to be answered. It is clear that further research is necessary to establish better *in vitro* and *in vivo* systems to study CHIKV infection and a consistent and reproducible picture of molecular immune response elicited against it, which in turn would pave the way to the discovery of markers that may be associated with disease morbidity and prognosis. Lastly, the treatment of CHIKVD is mostly symptomatic and no approved vaccine or antiviral drug currently exists. We believe that the development of safe and robust prevention and treatment approaches for CHIKV infection needs to be given top priority among researchers.

## Author contributions

All authors performed the literature search and wrote the initial draft of the manuscript. IT, ET, LA, and DY prepared the manuscript figures. LA, DY, and ÊB reviewed the initial draft and wrote the final version manuscript. NC revised the manuscript. All authors revised and agree with the final manuscript version.

### Conflict of interest statement

The authors declare that the research was conducted in the absence of any commercial or financial relationships that could be construed as a potential conflict of interest.

## References

[B1] Abdelnabi R. Neyts J. Delang L. (2015). Towards antivirals against chikungunya virus. Antiviral Res. 121, 59–68. 10.1016/j.antiviral.2015.06.017 26119058 PMC7113767

[B2] Agarwal A. Sharma A. K. Sukumaran D. Parida M. Dash P. K. (2016). Two novel epistatic mutations (E1:K211E and E2:V264A) in structural proteins of Chikungunya virus enhance fitness in *Aedes aegypti*. Virology 497, 59–68. 10.1016/j.virol.2016.06.025 27423270

[B3] Ahola T. Merits A. (2016). Functions of Chikungunya virus nonstructural proteins, in Chikungunya Virus: Advances in Biology, Pathogenesis, and Treatment, ed Okeoma C. M. (Cham: Springer International Publishing), 75–98.

[B4] Akahata W. Yang Z. Y. Andersen H. Sun S. Holdaway H. A. Kong W. P.. (2010). A virus-like particle vaccine for epidemic Chikungunya virus protects nonhuman primates against infection. Nat. Med. 16, 334–338. 10.1038/nm.2105 20111039 PMC2834826

[B5] Althouse B. M. Guerbois M. Cummings D. A. T. Diop O. M. Faye O. Faye A.. (2018). Role of monkeys in the sylvatic cycle of chikungunya virus in Senegal. Nat. Commun. 9, 1046. 10.1038/s41467-018-03332-7 29535306 PMC5849707

[B6] Amdekar S. Parashar D. Alagarasu K. (2017). Chikungunya virus-induced arthritis: role of host and viral factors in the pathogenesis. Viral Immunol. 30, 691–702. 10.1089/vim.2017.0052 28910194

[B7] Bao H. Ramanathan A. A. Kawalakar O. Sundaram S. G. Tingey C. Bian C. B.. (2013). Nonstructural protein 2 (nsP2) of Chikungunya virus (CHIKV) enhances protective immunity mediated by a CHIKV envelope protein expressing DNA Vaccine. Viral Immunol. 26, 75–83. 10.1089/vim.2012.0061 23409931 PMC4845693

[B8] Bartok B. Firestein G. S. (2010). Fibroblast-like synoviocytes: key effector cells in rheumatoid arthritis. Immunol. Rev. 233, 233–255. 10.1111/j.0105-2896.2009.00859 20193003 PMC2913689

[B9] Bocanegra C. Anton A. Sulleiro E. Pou D. Salvador F. Roure S.. (2016). Imported cases of Chikungunya in Barcelona in relation to the current American outbreak. J. Travel Med. 23:tav033. 10.1093/jtm/tav033 26984354

[B10] Borgherini G. Poubeau P. Jossaume A. Gouix A. Cotte L. Michault A.. (2008). Persistent arthralgia associated with chikungunya virus: a study of 88 adult patients on reunion island. Clin. Infect. Dis. 47, 469–475. 10.1086/590003 18611153

[B11] Brandler S. Ruffié C. Combredet C. Brault J. B. Najburg V. Prevost M. C.. (2013). A recombinant measles vaccine expressing chikungunya virus-like particles is strongly immunogenic and protects mice from lethal challenge with chikungunya virus. Vaccine 31, 3718–3725. 10.1016/j.vaccine.2013.05.086 23742993

[B12] Brighton S. W. Prozesky O. W. de la Harpe A. L. (1983). Chikungunya virus infection. A retrospective study of 107 cases. S. Afr. Med. J. 63, 313–315. 6298956

[B13] Broeckel R. Fox J. M. Haese N. Kreklywich C. N. Sukulpovi-Petty S. Legasse A.. (2017). Therapeutic administration of a recombinant human monoclonal antibody reduces the severity of chikungunya virus disease in rhesus macaques. PLoS Negl. Trop. Dis. 11:e0005637. 10.1371/journal.pntd.0005637 28628616 PMC5491320

[B14] Broeckel R. Haese N. Messaoudi I. Streblow D. N. (2015). Nonhuman primate models of Chikungunya virus infection and disease (CHIKV NHP Model). Pathogens 4, 662–681. 10.3390/pathogens4030662 26389957 PMC4584280

[B15] Burt F. J. Chen W. Miner J. J. Lenschow D. J. Merits A. Schnettler E.. (2017). Chikungunya virus: an update on the biology and pathogenesis of this emerging pathogen. Lancet Infect. Dis. 17, e107–e117. 10.1016/S1473-3099(16)30385-1 28159534

[B16] Campos G. S. Albuquerque Bandeira A. C. Diniz Rocha V. F. Dias J. P. Carvalho R. H. Sardi S. I. (2017). First detection of Chikungunya virus in breast milk. Pediatr. Infect. Dis. J. 36, 1015–1017. 10.1097/INF.0000000000001658 28650420

[B17] Chaaitanya I. K. Muruganandam N. Sundaram S. G. Kawalekar O. Sugunan A. P. Manimunda S. P.. (2011). Role of proinflammatory cytokines and chemokines in chronic arthropathy in CHIKV infection. Viral Immunol. 24, 265–271. 10.1089/vim.2010.0123 21830898

[B18] Chang L. J. Dowd K. A. Mendoza F. H. Saunders J. G. Sitar S. Plummer S. H.. (2014). Safety and tolerability of chikungunya virus-like particle vaccine in healthy adults: a phase 1 dose-escalation trial. Lancet 384, 2046–2052. 10.1016/S0140-6736(14)61185-5 25132507

[B19] Chattopadhyay A. Wang E. Seymour R. Weaver S. C. Rose J. K. (2013). A chimeric vesiculo/alphavirus is an effective alphavirus vaccine. J. Virol. 87, 395–402. 10.1128/JVI.01860-12 23077320 PMC3536361

[B20] Chen C. I. Clark D. C. Pesavento P. Lerche N. W. Luciw P. A. Reisen W. K.. (2010). Comparative pathogenesis of epidemic and enzootic Chikungunya viruses in a pregnant Rhesus macaque model. Am. J. Trop. Med. Hyg. 83, 1249–1258. 10.4269/ajtmh.2010.10-0290 21118930 PMC2990040

[B21] Chen W. Foo S. S. Taylor A. Lulla A. Merits A. Hueston L.. (2015). Bindarit, an inhibitor of monocyte chemotactic protein synthesis, protects against bone loss induced by chikungunya virus infection. J. Virol. 89, 581–593. 10.1128/JVI.02034-14 25339772 PMC4301140

[B22] Chen W. Foo S. S. Zaid A. Teng T. S. Herrero L. J. Wolf S.. (2017). Specific inhibition of NLRP3 in chikungunya disease reveals a role for inflammasomes in alphavirus-induced inflammation. Nat. Microbiol. 2, 1435–1445. 10.1038/s41564-017-0015-4 28848230

[B23] Chirathaworn C. Poovorawan Y. Lertmaharit S. Wuttirattanakowit N. (2013). Cytokine levels in patients with chikungunya virus infection. Asian Pac. J. Trop. Med. 6, 631–634. 10.1016/S1995-7645(13)60108-X 23790334

[B24] Chirathaworn C. Rianthavorn P. Wuttirattanakowit N. Poovorawan Y. (2010). Serum IL-18 and IL-18BP levels in patients with Chikungunya virus infection. Viral Immunol. 23, 113–117. 10.1089/vim.2009.0077 20121409

[B25] Chow A. Her Z. Ong E. K. Chen J. M. Dimatatac F. Kwek D. J.. (2011). Persistent arthralgia induced by Chikungunya virus infection is associated with interleukin-6 and granulocyte macrophage colony-stimulating factor. J. Infect. Dis. 203, 149–157. 10.1093/infdis/jiq042 21288813 PMC3071069

[B26] Chu H. Das S. C. Fuchs J. F. Suresh M. Weaver S. C. Stinchcomb D. T.. (2013). Deciphering the protective role of adaptive immunity to CHIKV/IRES a novel candidate vaccine against Chikungunya in the A129 mouse model. Vaccine 31, 3353–3360. 10.1016/j.vaccine.2013.05.059 23727003 PMC3731778

[B27] Chua C. L. Sam I. C. Chiam C. W. Chan Y. F. (2017). The neutralizing role of IgM during early Chikungunya virus infection. PLoS ONE 12:e0171989. 10.1371/journal.pone.0171989 28182795 PMC5300252

[B28] Chua H. H. Abdul Rashid K. Law W. C. Hamizah A. Chem Y. K. Khairul A. H.. (2010). A fatal case of chikungunya virus infection with liver involvement. Med. J. Malaysia 65, 83–84. 21265260

[B29] Clayton A. M. (2016). Monoclonal antibodies as prophylactic and therapeutic agents against Chikungunya virus. J. Infect. Dis. 214(Suppl. 5), S506–S509. 10.1093/infdis/jiw324 27920182 PMC5853615

[B30] Couderc T. Chrétien F. Schilte C. Disson O. Brigitte M. Guivel-Benhassine F.. (2008). A mouse model for Chikungunya: young age and inefficient type-I interferon signaling are risk factors for severe disease. PLoS Pathog. 4:e29. 10.1371/journal.ppat.0040029 18282093 PMC2242832

[B31] Das T. Hoarau J. J. Jaffar Bandjee M. C. Maquart M. Gasque P. (2015). Multifaceted innate immune responses engaged by astrocytes, microglia and resident dendritic cells against Chikungunya neuroinfection. J. Gen. Virol. 96(Pt 2), 294–310. 10.1099/vir.0.071175-0 25351727

[B32] Dash P. K. Tiwari M. Santhosh S. R. Parida M. Lakshmana Rao P. V. (2008). RNA interference mediated inhibition of Chikungunya virus replication in mammalian cells. Biochem. Biophys. Res. Commun. 376, 718–722. 10.1016/j.bbrc.2008.09.040 18805396

[B33] DeZure A. D. Berkowitz N. M. Graham B. S. Ledgerwood J. E. (2016). Whole-inactivated and virus-like particle vaccine strategies for Chikungunya virus. J. Infect. Dis. 214(Suppl. 5), S497–S499. 10.1093/infdis/jiw352 27920180 PMC5137244

[B34] Diallo D. Sall A. A. Buenemann M. Chen R. Faye O. Diagne C. T.. (2012). Landscape ecology of sylvatic chikungunya virus and mosquito vectors in southeastern Senegal. PLoS Negl. Trop. Dis. 6:e1649. 10.1371/journal.pntd.0001649 22720097 PMC3373654

[B35] Dupuis-Maguiraga L. Noret M. Brun S. Le Grand R. Gras G. Roques P. (2012). Chikungunya disease: infection-associated markers from the acute to the chronic phase of arbovirus-induced arthralgia. PLoS Negl. Trop. Dis. 6:e1446. 10.1371/journal.pntd.0001446 22479654 PMC3313943

[B36] Economopoulou A. Dominguez M. Helynck B. Sissoko D. Wichmann O. Quenel P.. (2009). Atypical Chikungunya virus infections: clinical manifestations, mortality and risk factors for severe disease during the 2005–2006 outbreak on Reunion. Epidemiol. Infect. 137, 534–541. 10.1017/S0950268808001167 18694529

[B37] Edelman R. Tacket C. O. Wasserman S. S. Bodison S. A. Perry J. G. Mangiafico J. A. (2000). Phase II safety and immunogenicity study of live chikungunya virus vaccine TSI-GSD-218. Am. J. Trop. Med. Hyg. 62, 681–685. 11304054 10.4269/ajtmh.2000.62.681

[B38] Edwards T. Del Carmen Castillo Signor L. Williams C. Larcher C. Espinel M. Theaker J.. (2017). Analytical and clinical performance of a Chikungunya qRT-PCR for Central and South America. Diagn. Microbiol. Infect. Dis. 89, 35–39. 10.1016/j.diagmicrobio.2017.06.001 28633900 PMC5560405

[B39] Erasmus J. H. Auguste A. J. Kaelber J. T. Luo H. Rossi S. L. Fenton K.. (2017). A chikungunya fever vaccine utilizing an insect-specific virus platform. Nat. Med. 23, 192–199. 10.1038/nm.4253 27991917 PMC5296253

[B40] Firth A. E. Chung B. Y. Fleeton M. N. Atkins J. F. (2008). Discovery of frameshifting in Alphavirus 6K resolves a 20-year enigma. Virol. J. 5, 108. 10.1186/1743-422X-5-108 18822126 PMC2569925

[B41] Fong R. H. Banik S. S. Mattia K. Barnes T. Tucker D. Liss N.. (2014). Exposure of epitope residues on the outer face of the chikungunya virus envelope trimer determines antibody neutralizing efficacy. J. Virol. 88, 14364–14379. 10.1128/JVI.01943-14 25275138 PMC4249124

[B42] Fox J. M. Diamond M. S. (2016). Immune-mediated protection and pathogenesis of Chikungunya virus. J. Immunol. 197, 4210–4218. 10.4049/jimmunol.1601426 27864552 PMC5120763

[B43] García-Arriaza J. Cepeda V. Hallengärd D. Sorzano C. Ó Kümmerer B. M. Liljeström P.. (2014). A novel poxvirus-based vaccine, MVA-CHIKV, is highly immunogenic and protects mice against chikungunya infection. J. Virol. 88, 3527–3547. 10.1128/JVI.03418-13 24403588 PMC3957920

[B44] Gardner J. Anraku I. Le T. T. Larcher T. Major L. Roques P.. (2010). Chikungunya virus arthritis in adult wild-type mice. J. Virol. 84, 8021–8032. 10.1128/JVI.02603-09 20519386 PMC2916516

[B45] Gérardin P. Barau G. Michault A. Bintner M. Randrianaivo H. Choker G.. (2008). Multidisciplinary prospective study of mother-to-child chikungunya virus infections on the island of La Reunion. PLoS Med. 5:e60. 10.1371/journal.pmed.0050060 18351797 PMC2267812

[B46] Gérardin P. Fianu A. Malvy D. Mussard C. Boussaïd K. Rollot O.. (2011). Perceived morbidity and community burden after a Chikungunya outbreak: the TELECHIK survey, a population-based cohort study. BMC Med. 9:5. 10.1186/1741-7015-9-5 21235760 PMC3029216

[B47] Go Y. Y. Balasuriya U. B. Lee C. K. (2014). Zoonotic encephalitides caused by arboviruses: transmission and epidemiology of alphaviruses and flaviviruses. Clin. Exp. Vaccine Res. 3, 58–77. 10.7774/cevr.2014.3.1.58 24427764 PMC3890452

[B48] Haese N. N. Broeckel R. M. Hawman D. W. Heise M. T. Morrison T. E. Streblow D. N. (2016). Animal models of Chikungunya virus infection and disease. J. Infect. Dis. 214(Suppl. 5), S482–S487. 10.1093/infdis/jiw284 27920178 PMC5137241

[B49] Haist K. C. Burrack K. S. Davenport B. J. Morrison T. E. (2017). Inflammatory monocytes mediate control of acute alphavirus infection in mice. PLoS Pathog. 13:e1006748. 10.1371/journal.ppat.1006748 29244871 PMC5747464

[B50] Hallengärd D. Kakoulidou M. Lulla A. Kümmerer B. M. Johansson D. X. Mutso M.. (2014). Novel attenuated Chikungunya vaccine candidates elicit protective immunity in C57BL/6 mice. J. Virol. 88, 2858–2866. 10.1128/JVI.03453-13 24371047 PMC3958085

[B51] Harrison V. R. Eckels K. H. Bartelloni P. J. Hampton C. (1971). Production and evaluation of a formalin-killed Chikungunya vaccine. J. Immunol. 107, 643–647. 4999088

[B52] Hawman D. W. Stoermer K. A. Montgomery S. A. Pal P. Oko L. Diamond M. S.. (2013). Chronic joint disease caused by persistent Chikungunya virus infection is controlled by the adaptive immune response. J. Virol. 87, 13878–13888. 10.1128/JVI.02666-13 24131709 PMC3838294

[B53] Her Z. Malleret B. Chan M. Ong E. K. Wong S. C. Kwek D. J.. (2010). Active infection of human blood monocytes by Chikungunya virus triggers an innate immune response. J. Immunol. 184, 5903–5913. 10.4049/jimmunol.0904181 20404274

[B54] Hoarau J. J. Jaffar Bandjee M. C. Krejbich Trotot P. Das T. Li-Pat-Yuen G. Dassa B.. (2010). Persistent chronic inflammation and infection by Chikungunya arthritogenic alphavirus in spite of a robust host immune response. J. Immunol. 184, 5914–5927. 10.4049/jimmunol.0900255 20404278

[B55] Hoz J. M. Bayona B. Viloria S. Accini J. L. Juan-Vergara H. S. Viasus D. (2015). Fatal cases of Chikungunya virus infection in Colombia: diagnostic and treatment challenges. J. Clin. Virol. 69, 27–29. 10.1016/j.jcv.2015.05.021 26209372

[B56] Huang B. Pyke A. T. McMahon J. Warrilow D. (2017). Complete coding sequence of a case of Chikungunya virus imported into Australia. Genome Announc. 5:e00310-17. 10.1128/genomeA.00310-17 28495775 PMC5427210

[B57] Huits R. De Kort J. Van Den Berg R. Chong L. Tsoumanis A. Eggermont K.. (2018). Chikungunya virus infection in Aruba: diagnosis, clinical features and predictors of post-chikungunya chronic polyarthralgia. PLoS ONE 13:e0196630. 10.1371/journal.pone.0196630 29709007 PMC5927412

[B58] Jain J. Okabayashi T. Kaur N. Nakayama E. Shioda T. Gaind R.. (2018). Evaluation of an immunochromatography rapid diagnosis kit for detection of chikungunya virus antigen in India, a dengue-endemic country. Virol. J. 15, 84. 10.1186/s12985-018-1000-0 29751761 PMC5948817

[B59] Jamil K. M. Khakoo S. I. (2011). KIR/HLA interactions and pathogen immunity. J. Biomed. Biotechnol. 2011, 298348. 10.1155/2011/298348 21629750 PMC3100571

[B60] Javelle E. Ribera A. Degasne I. Gaüzère B. A. Marimoutou C. Simon F. (2015). Specific management of post-chikungunya rheumatic disorders: a retrospective study of 159 cases in Reunion Island from 2006–2012. PLoS Negl. Trop. Dis. 9:e0003603. 10.1371/journal.pntd.0003603 25760632 PMC4356515

[B61] Johnson B. W. Goodman C. H. Holloway K. de Salazar P. M. Valadere A. M. Drebot M. A. (2016a). Evaluation of commercially available Chikungunya virus immunoglobulin M detection assays. Am. J. Trop. Med. Hyg. 95, 182–192. 10.4269/ajtmh.16-0013 26976887 PMC4944686

[B62] Johnson B. W. Russell B. J. Goodman C. H. (2016b). Laboratory diagnosis of Chikungunya virus infections and commercial sources for diagnostic assays. J. Infect. Dis. 214(Suppl. 5), S471–S474. 10.1093/infdis/jiw274 27920176 PMC5657184

[B63] Jose J. Snyder J. E. Kuhn R. J. (2009). A structural and functional perspective of alphavirus replication and assembly. Fut. Microbiol. 4, 837–856. 10.2217/fmb.09.59 19722838 PMC2762864

[B64] Josseran L. Paquet C. Zehgnoun A. Caillere N. Le Tertre A. Solet J. L.. (2006). Chikungunya disease outbreak, Reunion Island. Emerg. Infect. Dis. 12, 1994–1995. 10.3201/eid1212.060710 17354339 PMC3291364

[B65] Kam Y. W. Lee W. W. Simarmata D. Harjanto S. Teng T. S. Tolou H.. (2012a). Longitudinal analysis of the human antibody response to Chikungunya virus infection: implications for serodiagnosis and vaccine development. J. Virol. 86, 13005–13015. 10.1128/JVI.01780-12 23015702 PMC3497641

[B66] Kam Y. W. Simarmata D. Chow A. Her Z. Teng T. S. Ong E. K.. (2012b). Early appearance of neutralizing immunoglobulin G3 antibodies is associated with chikungunya virus clearance and long-term clinical protection. J. Infect. Dis. 205, 1147–1154. 10.1093/infdis/jis033 22389226 PMC3295607

[B67] Kashyap R. S. Morey S. H. Ramteke S. S. Chandak N. H. Parida M. Deshpande P. S.. (2010). Diagnosis of Chikungunya fever in an Indian population by an indirect enzyme-linked immunosorbent assay protocol based on an antigen detection assay: a prospective cohort study. Clin. Vaccine Immunol. 17, 291–297. 10.1128/CVI.00326-09 20007365 PMC2815524

[B68] Kelvin A. A. Banner D. Silvi G. Moro M. L. Spataro N. Gaibani P.. (2011). Inflammatory cytokine expression is associated with chikungunya virus resolution and symptom severity. PLoS Negl. Trop. Dis. 5:e1279. 10.1371/journal.pntd.0001279 21858242 PMC3156690

[B69] Kendrick K. Stanek D. Blackmore C. Centers for Disease Control and Prevention (CDC). (2014). Notes from the field: transmission of chikungunya virus in the continental United States–Florida, 2014. MMWR Morb. Mortal. Wkly. Rep. 63, 1137. 25474035 PMC4584604

[B70] Khan A. H. Morita K. Parquet Md Mdel C. Hasebe F. Mathenge E. G. Igarashi A. (2002). Complete nucleotide sequence of chikungunya virus and evidence for an internal polyadenylation site. J. Gen. Virol. 83(Pt 12), 3075–3084. 10.1099/0022-1317-83-12-3075 12466484

[B71] Khan M. Dhanwani R. Rao P. V. Parida M. (2012). Subunit vaccine formulations based on recombinant envelope proteins of Chikungunya virus elicit balanced Th1/Th2 response and virus-neutralizing antibodies in mice. Virus Res. 167, 236–246. 10.1016/j.virusres.2012.05.004 22610133

[B72] Kumar M. Sudeep A. B. Arankalle V. A. (2012a). Evaluation of recombinant E2 protein-based and whole-virus inactivated candidate vaccines against chikungunya virus. Vaccine 30, 6142–6149. 10.1016/j.vaccine.2012.07.072 22884660

[B73] Kumar S. Jaffar-Bandjee M. C. Giry C. Connen de Kerillis L. Merits A. Gasque P.. (2012b). Mouse macrophage innate immune response to Chikungunya virus infection. Virol. J. 9, 313. 10.1186/1743-422X-9-313 23253140 PMC3577478

[B74] Labadie K. Larcher T. Joubert C. Mannioui A. Delache B. Brochard P.. (2010). Chikungunya disease in nonhuman primates involves long-term viral persistence in macrophages. J. Clin. Invest. 120, 894–906. 10.1172/JCI40104 20179353 PMC2827953

[B75] Lam S. Chen H. Chen C. K. Min N. Chu J. J. (2015). Antiviral phosphorodiamidate morpholino oligomers are protective against Chikungunya virus infection on cell-based and murine models. Sci. Rep. 5, 12727. 10.1038/srep12727 26224141 PMC4649900

[B76] Lang P. O. Loulergue P. Aspinall R. (2017). Chikungunya virus infection: why should U.S. Geriatricians be aware of it? J. Am. Geriatr. Soc. 65, 2529–2534. 10.1111/jgs.15104 28940385

[B77] Langsjoen R. M. Haller S. L. Roy C. J. Vinet-Oliphant H. Bergren N. A. Erasmus J. H.. (2018). Chikungunya virus strains show lineage-specific variations in virulence and cross-protective ability in murine and nonhuman primate models. MBio 9:e02449–17. 10.1128/mBio.02449-17 29511072 PMC5844994

[B78] Laoprasopwattana K. Suntharasaj T. Petmanee P. Suddeaugrai O. Geater A. (2015). Chikungunya and dengue virus infections during pregnancy: seroprevalence, seroincidence and maternal–fetal transmission, southern Thailand, 2009–2010. Epidemiol. Infect. 144, 381–388. 10.1017/s0950268815001065 26113247

[B79] Leparc-Goffart I. Nougairede A. Cassadou S. Prat C. de Lamballerie X. (2014). Chikungunya in the Americas. Lancet 383, 514. 10.1016/S0140-6736(14)60185-9 24506907

[B80] Ljungberg K. Kümmerer B. M. Roques P. Esteban M. Merits A. Liljeström P. (2016). Vaccines against Chikungunya virus infection, in Chikungunya Virus, ed Okeoma C. (Cham: Springer), 45–62. 10.1007/978-3-319-42958-8_4

[B81] Lohachanakul J. Phuklia W. Thannagith M. Thonsakulprasert T. Ubol S. (2012). High concentrations of circulating interleukin-6 and monocyte chemotactic protein-1 with low concentrations of interleukin-8 were associated with severe chikungunya fever during the 2009–2010 outbreak in Thailand. Microbiol. Immunol. 56, 134–138. 10.1111/j.1348-0421.2011.00417.x 22188545

[B82] Long K. M. Ferris M. T. Whitmore A. C. Montgomery S. A. Thurlow L. R. McGee C. E.. (2016). Gammadelta T cells play a protective role in Chikungunya virus-induced disease. J. Virol. 90, 433–443. 10.1128/JVI.02159-15 26491151 PMC4702549

[B83] Long K. M. Heise M. T. (2015). Protective and pathogenic responses to Chikungunya virus infection. Curr. Trop. Med. Rep. 2, 13–21. 10.1007/s40475-015-0037-z 26366337 PMC4564112

[B84] Long K. M. Whitmore A. C. Ferris M. T. Sempowski G. D. McGee C. Trollinger B.. (2013). Dendritic cell immunoreceptor regulates Chikungunya virus pathogenesis in mice. J. Virol. 87, 5697–5706. 10.1128/JVI.01611-12 23487448 PMC3648201

[B85] Lourenço-de-Oliveira R. Failloux A. B. (2017). High risk for chikungunya virus to initiate an enzootic sylvatic cycle in the tropical Americas. PLoS Negl. Trop. Dis. 11:e0005698. 10.1371/journal.pntd.0005698 28662031 PMC5507584

[B86] Lum F. M. Couderc T. Chia B. S. Ong R. Y. Her Z. Chow A.. (2018). Antibody-mediated enhancement aggravates chikungunya virus infection and disease severity. Sci. Rep. 8, 1860. 10.1038/s41598-018-20305-4 29382880 PMC5789897

[B87] Lum F. M. Ng L. F. (2015). Cellular and molecular mechanisms of chikungunya pathogenesis. Antiviral Res. 120, 165–174. 10.1016/j.antiviral.2015.06.009 26092642

[B88] Lum F. M. Teo T. H. Lee W. W. Kam Y. W. Rénia L. Ng L. F. (2013). An essential role of antibodies in the control of Chikungunya virus infection. J. Immunol. 190, 6295–6302. 10.4049/jimmunol.1300304 23670192 PMC3677171

[B89] Madariaga M. Ticona E. Resurrecion C. (2016). Chikungunya: bending over the Americas and the rest of the world. Braz. J. Infect. Dis. 20, 91–98. 10.1016/j.bjid.2015.10.004 26707971 PMC9425360

[B90] Mallilankaraman K. Shedlock D. J. Bao H. Kawalekar O. U. Fagone P. Ramanathan A. A.. (2011). A DNA vaccine against chikungunya virus is protective in mice and induces neutralizing antibodies in mice and nonhuman primates. PLoS Negl. Trop. Dis. 5:e928. 10.1371/journal.pntd.0000928 21264351 PMC3019110

[B91] Melton J. V. Ewart G. D. Weir R. C. Board P. G. Lee E. Gage P. W. (2002). Alphavirus 6K proteins form ion channels. J. Biol. Chem. 277, 46923–46931. 10.1074/jbc.M207847200 12228229

[B92] Messaoudi I. Vomaske J. Totonchy T. Kreklywich C. N. Haberthur K. Springgay L.. (2013). Chikungunya virus infection results in higher and persistent viral replication in aged rhesus macaques due to defects in anti-viral immunity. PLoS Negl. Trop. Dis. 7:e2343. 10.1371/journal.pntd.0002343 23936572 PMC3723534

[B93] Metz S. W. Geertsema C. Martina B. E. Andrade P. Heldens J. G. van Oers M. M.. (2011). Functional processing and secretion of Chikungunya virus E1 and E2 glycoproteins in insect cells. Virol. J. 8, 353. 10.1186/1743-422X-8-353 21762510 PMC3162542

[B94] Metz S. W. Martina B. E. van den Doel P. Geertsema C. Osterhaus A. D. Vlak J. M.. (2013). Chikungunya virus-like particles are more immunogenic in a lethal AG129 mouse model compared to glycoprotein E1 or E2 subunits. Vaccine 31, 6092–6096. 10.1016/j.vaccine.2013.09.045 24099875

[B95] Metz S. W. Pijlman G. P. (2016). Function of Chikungunya virus structural proteins in Chikungunya Virus, ed Okeoma C. (Cham: Springer), 63–74. 10.1007/978-3-319-42958-8_5

[B96] Miner J. J. Aw-Yeang H. X. Fox J. M. Taffner S. Malkova O. N. Oh S. T.. (2015). Chikungunya viral arthritis in the United States: a mimic of seronegative rheumatoid arthritis. Arthritis Rheumatol. 67, 1214–1220. 10.1002/art.39027 25605621 PMC4591551

[B97] Miner J. J. Cook L. E. Hong J. P. Smith A. M. Richner J. M. Shimak R. M.. (2017). Therapy with CTLA4-Ig and an antiviral monoclonal antibody controls chikungunya virus arthritis. Sci. Transl. Med. 9:eaah343. 10.1126/scitranslmed.aah3438 28148840 PMC5448557

[B98] Moro M. L. Grilli E. Corvetta A. Silvi G. Angelini R. Mascella F.. (2012). Long-term chikungunya infection clinical manifestations after an outbreak in Italy: a prognostic cohort study. J. Infect. 65, 165–172. 10.1016/j.jinf.2012.04.005 22522292

[B99] Mourya D. T. Mishra A. C. (2006). Chikungunya fever. Lancet 368, 186–187. 10.1016/S0140-6736(06)69017-X 16844472

[B100] Muthumani K. Block P. Flingai S. Muruganantham N. Chaaithanya I. K. Tingey C.. (2016). Rapid and long-term immunity elicited by DNA-encoded antibody prophylaxis and DNA vaccination against Chikungunya virus. J. Infect. Dis. 214, 369–378. 10.1093/infdis/jiw111 27001960 PMC4936642

[B101] Nair P. M. (2008). Chikungunya in neonates. Indian Pediatr. 45, 605. 18695288

[B102] Nayak T. K. Mamidi P. Kumar A. Singh L. P. Sahoo S. S. Chattopadhyay S.. (2017). Regulation of viral replication, apoptosis and pro-inflammatory responses by 17-AAG during Chikungunya virus infection in macrophages. Viruses 9:3. 10.3390/v9010003 28067803 PMC5294972

[B103] Ng L. F. Chow A. Sun Y. J. Kwek D. J. Lim P. L. Dimatatac F.. (2009). IL-1beta, IL-6, and RANTES as biomarkers of Chikungunya severity. PLoS ONE 4:e4261. 10.1371/journal.pone.0004261 19156204 PMC2625438

[B104] Nielsen C. M. White M. J. Goodier M. R. Riley E. M. (2013). Functional significance of CD57 expression on human NK cells and relevance to disease. Front. Immunol. 4:422. 10.3389/fimmu.2013.00422 24367364 PMC3856678

[B105] Noret M. Herrero L. Rulli N. Rolph M. Smith P. N. Li R. W.. (2012). Interleukin 6, RANKL, and osteoprotegerin expression by chikungunya virus-infected human osteoblasts. J. Infect. Dis. 206, 455–457: 457–459. 10.1093/infdis/jis368 22634878

[B106] Pal P. Dowd K. A. Brien J. D. Edeling M. A. Gorlatov S. Johnson S.. (2013). Development of a highly protective combination monoclonal antibody therapy against Chikungunya virus. PLoS Pathog. 9:e1003312. 10.1371/journal.ppat.1003312 23637602 PMC3630103

[B107] Pal P. Fox J. M. Hawman D. W. Huang Y. J. Messaoudi I. Kreklywich C.. (2014). Chikungunya viruses that escape monoclonal antibody therapy are clinically attenuated, stable, and not purified in mosquitoes. J. Virol. 88, 8213–8226. 10.1128/JVI.01032-14 24829346 PMC4135940

[B108] Pan American Health Organization (2011). Preparedness and Response for Chikungunya Virus: Introduction in the Americas. Washington, DC: PAHO.

[B109] Parashar D. Paingankar M. S. Kumar S. Gokhale M. D. Sudeep A. B. Shinde S. B.. (2013). Administration of E2 and NS1 siRNAs inhibit chikungunya virus replication *in vitro* and protects mice infected with the virus. PLoS Negl. Trop. Dis. 7:e2405. 10.1371/journal.pntd.0002405 24040429 PMC3764232

[B110] Partidos C. D. Paykel J. Weger J. Borland E. M. Powers A. M. Seymour R.. (2012). Cross-protective immunity against O'nyong-nyong virus afforded by a novel recombinant chikungunya vaccine. Vaccine 30, 4638–4643. 10.1016/j.vaccine.2012.04.099 22583812 PMC3372665

[B111] Pastorino B. Bessaud M. Grandadam M. Murri S. Tolou H. J. Peyrefitte C. N. (2005). Development of a TaqMan RT-PCR assay without RNA extraction step for the detection and quantification of African Chikungunya viruses. J. Virol. Methods 124, 65–71. 10.1016/j.jviromet.2004.11.002 15664052

[B112] Patil D. R. Hundekar S. L. Arankalle V. A. (2012). Expression profile of immune response genes during acute myopathy induced by chikungunya virus in a mouse model. Microbes Infect. 14, 457–469. 10.1016/j.micinf.2011.12.008 22230246

[B113] Pegram H. J. Andrews D. M. Smyth M. J. Darcy P. K. Kershaw M. H. (2011). Activating and inhibitory receptors of natural killer cells. Immunol. Cell Biol. 89, 216–224. 10.1038/icb.2010.78 20567250

[B114] Petitdemange C. Becquart P. Wauquier N. Béziat V. Debré P. Leroy E. M.. (2011). Unconventional repertoire profile is imprinted during acute chikungunya infection for natural killer cells polarization toward cytotoxicity. PLoS Pathog. 7:e1002268. 10.1371/journal.ppat.1002268 21966274 PMC3178577

[B115] Petitdemange C. Wauquier N. Devilliers H. Yssel H. Mombo I. Caron M.. (2016). Longitudinal analysis of natural killer cells in dengue virus-infected patients in comparison to Chikungunya and Chikungunya/Dengue virus-infected patients. PLoS Negl. Trop. Dis. 10:e0004499. 10.1371/journal.pntd.0004499 26938618 PMC4777550

[B116] Petitdemange C. Wauquier N. Jacquet J. M. Theodorou I. Leroy E. Vieillard V. (2014). Association of HLA class-I and inhibitory KIR genotypes in Gabonese patients infected by Chikungunya or Dengue type-2 viruses. PLoS ONE 9:e108798. 10.1371/journal.pone.0108798 25264760 PMC4181859

[B117] Pfeffer M. Linssen B. Parke M. D. Kinney R. M. (2002). Specific detection of chikungunya virus using a RT-PCR/nested PCR combination. J. Vet. Med. B Infect. Dis. Vet Public Health 49, 49–54. 10.1046/j.1439-0450.2002.00535.x 11911593

[B118] Phuklia W. Kasisith J. Modhiran N. Rodpai E. Thannagith M. Thongsakulprasert T.. (2013). Osteoclastogenesis induced by CHIKV-infected fibroblast-like synoviocytes: a possible interplay between synoviocytes and monocytes/macrophages in CHIKV-induced arthralgia/arthritis. Virus Res. 177, 179–188. 10.1016/j.virusres.2013.08.011 24012515

[B119] Plante K. Wang E. Partidos C. D. Weger J. Gorchakov R. Tsetsarkin K.. (2011). Novel chikungunya vaccine candidate with an IRES-based attenuation and host range alteration mechanism. PLoS Pathog. 7:e1002142. 10.1371/journal.ppat.1002142 21829348 PMC3145802

[B120] Poo Y. S. Nakaya H. Gardner J. Larcher T. Schroder W. A. Le T. T.. (2014). CCR2 deficiency promotes exacerbated chronic erosive neutrophil-dominated chikungunya virus arthritis. J. Virol. 88, 6862–6872. 10.1128/JVI.03364-13 24696480 PMC4054367

[B121] Powers A. M. (2018). Vaccine and therapeutic options to control Chikungunya virus. Clin. Microbiol. Rev. 31:e00104–16. 10.1128/CMR.00104-16 29237708 PMC5740971

[B122] Powers A. M. Brault A. C. Tesh R. B. Weaver S. C. (2000). Re-emergence of Chikungunya and O'nyong-nyong viruses: evidence for distinct geographical lineages and distant evolutionary relationships. J. Gen. Virol. 81(Pt 2), 471–479. 10.1099/0022-1317-81-2-471 10644846

[B123] Prince H. E. Seaton B. L. Matud J. L. Batterman H. J. (2015). Chikungunya virus RNA and antibody testing at a National Reference Laboratory since the emergence of Chikungunya virus in the Americas. Clin. Vaccine Immunol. 22, 291–297. 10.1128/CVI.00720-14 25540275 PMC4340891

[B124] Rajapakse S. Rodrigo C. Rajapakse A. (2010). Atypical manifestations of chikungunya infection. Trans. R. Soc. Trop. Med. Hyg. 104, 89–96. 10.1016/j.trstmh.2009.07.031 19716149

[B125] Ramful D. Carbonnier M. Pasquet M. Bouhmani B. Ghazouani J. Noormahomed T.. (2007). Mother-to-child transmission of Chikungunya virus infection. Pediatr. Infect. Dis. J. 26, 811–815. 10.1097/INF.0b013e3180616d4f 17721376

[B126] Ramsauer K. Schwameis M. Firbas C. Müllner M. Putnak R. J. Thomas S. J.. (2015). Immunogenicity, safety, and tolerability of a recombinant measles-virus-based chikungunya vaccine: a randomised, double-blind, placebo-controlled, active-comparator, first-in-man trial. Lancet Infect. Dis. 15, 519–527. 10.1016/S1473-3099(15)70043-5 25739878

[B127] Rao G. Khan Y. Z. Chitnis D. S. (2008). Chikungunya infection in neonates. Indian Pediatr. 45, 240–242. 18367775

[B128] Ravichandran R. Manian M. (2008). Ribavirin therapy for Chikungunya arthritis. J. Infect. Dev. Ctries 2, 140–142. 10.3855/jidc.286 19738340

[B129] Reddy V. Desai A. Krishna S. S. Vasanthapuram R. (2017). Molecular mimicry between chikungunya virus and host components: a possible mechanism for the arthritic manifestations. PLoS Negl. Trop. Dis. 11:e0005238. 10.1371/journal.pntd.0005238 28125580 PMC5268390

[B130] Reddy V. Mani R. S. Desai A. Ravi V. (2014). Correlation of plasma viral loads and presence of Chikungunya IgM antibodies with cytokine/chemokine levels during acute Chikungunya virus infection. J. Med. Virol. 86, 1393–1401. 10.1002/jmv.23875 24523146

[B131] Robin S. Ramful D. Le Seach F. Jaffar-Bandjee M. C. Rigou G. Alessandri J. L. (2008). Neurologic manifestations of pediatric chikungunya infection. J. Child. Neurol. 23, 1028–1035. 10.1177/0883073808314151 18287573

[B132] Robin S. Ramful D. Zettor J. Benhamou L. Jaffar-Bandjee M. C. Rivière J. P.. (2010). Severe bullous skin lesions associated with Chikungunya virus infection in small infants. Eur. J. Pediatr. 169, 67–72. 10.1007/s00431-009-0986-0 19401826

[B133] Robinson M. C. (1955). An epidemic of virus disease in Southern Province, Tanganyika Territory, in 1952–53. I. Clinical features. Trans. R. Soc. Trop. Med. Hyg. 49, 28–32. 14373834 10.1016/0035-9203(55)90080-8

[B134] Rodriguez-Morales A. J. Cardona-Ospina J. A. Fernanda Urbano-Garzón S. Sebastian Hurtado-Zapata J. (2016). Prevalence of post-Chikungunya infection chronic inflammatory arthritis: a systematic review and meta-analysis. Arthritis Care Res. (Hoboken) 68, 1849–1858. 10.1002/acr.22900 27015439

[B135] Roosenhoff R. Anfasa F. Martina B. (2016). The pathogenesis of chronic chikungunya: evolving concepts. Fut. Virol. 11, 61–77. 10.2217/fvl.15.107

[B136] Roques P. Ljungberg K. Kümmerer B. M. Gosse L. Dereuddre-Bosquet N. Tchitchek N.. (2017). Attenuated and vectored vaccines protect nonhuman primates against Chikungunya virus. JCI Insight 2:e83527. 10.1172/jci.insight.83527 28352649 PMC5358498

[B137] Roy C. J. Adams A. P. Wang E. Plante K. Gorchakov R. Seymour R. L.. (2014). Chikungunya vaccine candidate is highly attenuated and protects nonhuman primates against telemetrically monitored disease following a single dose. J. Infect. Dis. 209, 1891–1899. 10.1093/infdis/jiu014 24403555 PMC4038141

[B138] Rudd P. A. Raphael A. P. Yamada M. Nufer K. L. Gardner J. Le T. T.. (2015). Effective cutaneous vaccination using an inactivated chikungunya virus vaccine delivered by Foroderm. Vaccine 33, 5172–5180. 10.1016/j.vaccine.2015.07.099 26296498

[B139] Rupp J. C. Sokoloski K. J. Gebhart N. N. Hardy R. W. (2015). Alphavirus RNA synthesis and non-structural protein functions. J. Gen. Virol. 96, 2483–2500. 10.1099/jgv.0.000249 26219641 PMC4635493

[B140] Sam I. C. Kümmerer B. M. Chan Y. F. Roques P. Drosten C. AbuBakar S. (2015). Updates on chikungunya epidemiology, clinical disease, and diagnostics. Vector Borne Zoonotic Dis. 15, 223–230. 10.1089/vbz.2014.1680 25897809

[B141] Saraswat S. Athmaram T. N. Parida M. Agarwal A. Saha A. Dash P. K. (2016). Expression and characterization of yeast derived Chikungunya virus like particles (CHIK-VLPs) and its evaluation as a potential vaccine candidate. PLoS Negl. Trop. Dis. 10:e0004782. 10.1371/journal.pntd.0004782 27399001 PMC4939942

[B142] Schett G. (2007). Cells of the synovium in rheumatoid arthritis. Osteoclasts. Arthritis Res. Ther. 9, 203. 10.1186/ar2110 17316459 PMC1860063

[B143] Schilte C. Staikowsky F. Couderc T. Madec Y. Carpentier F. Kassab S.. (2013). Chikungunya virus-associated long-term arthralgia: a 36-month prospective longitudinal study. PLoS Negl. Trop. Dis. 7:e2137. 10.1371/journal.pntd.0002137 23556021 PMC3605278

[B144] Schuffenecker I. Iteman I. Michault A. Murri S. Frangeul L. Vaney M. C.. (2006). Genome microevolution of chikungunya viruses causing the Indian Ocean outbreak. PLoS Med. 3:e263. 10.1371/journal.pmed.0030263 16700631 PMC1463904

[B145] Schwartz O. Albert M. L. (2010). Biology and pathogenesis of chikungunya virus. Nat. Rev. Microbiol. 8, 491–500. 10.1038/nrmicro2368 20551973

[B146] Sebastian M. R. Lodha R. Kabra S. K. (2009). Chikungunya infection in children. Indian J. Pediatr. 76, 185–189. 10.1007/s12098-009-0049-6 19330307

[B147] Selvarajah S. Sexton N. R. Kahle K. M. Fong R. H. Mattia K. A. Gardner J.. (2013). A neutralizing monoclonal antibody targeting the acid-sensitive region in chikungunya virus E2 protects from disease. PLoS Negl. Trop. Dis. 7:e2423. 10.1371/journal.pntd.0002423 24069479 PMC3772074

[B148] Silva L. A. Dermody T. S. (2017). Chikungunya virus: epidemiology, replication, disease mechanisms, and prospective intervention strategies. J. Clin. Invest. 127, 737–749. 10.1172/JCI84417 28248203 PMC5330729

[B149] Simarmata D. Ng D. C. Kam Y. W. Lee B. Sum M. S. Her Z.. (2016). Early clearance of Chikungunya virus in children is associated with a strong innate immune response. Sci. Rep. 6, 26097. 10.1038/srep26097 27180811 PMC4867653

[B150] Singh A. Kumar A. Yadav R. Uversky V. N. Giri R. (2018). Deciphering the dark proteome of Chikungunya virus. Sci. Rep. 8, 5822. 10.1038/s41598-018-23969-0 29643398 PMC5895634

[B151] Singh R. K. Tiwari S. Mishra V. K. Tiwari R. Dhole T. N. (2012). Molecular epidemiology of Chikungunya virus: mutation in E1 gene region. J. Virol. Methods 185, 213–220. 10.1016/j.jviromet.2012.07.001 22782121

[B152] Smith S. A. Silva L. A. Fox J. M. Flyak A. I. Kose N. Sapparapu G.. (2015). Isolation and characterization of broad and ultrapotent human monoclonal antibodies with therapeutic activity against Chikungunya virus. Cell Host Microbe 18, 86–95. 10.1016/j.chom.2015.06.009 26159721 PMC4501771

[B153] Snyder A. J. Mukhopadhyay S. (2012). The alphavirus E3 glycoprotein functions in a clade-specific manner. J. Virol. 86, 13609–13620. 10.1128/JVI.01805-12 23035234 PMC3503070

[B154] Snyder J. E. Kulcsar K. A. Schultz K. L. Riley C. P. Neary J. T. Marr S.. (2013). Functional characterization of the alphavirus TF protein. J. Virol. 87, 8511–8523. 10.1128/JVI.00449-13 23720714 PMC3719798

[B155] Solignat M. Gay B. Higgs S. Briant L. Devaux C. (2009). Replication cycle of chikungunya: a re-emerging arbovirus. Virology 393, 183–197. 10.1016/j.virol.2009.07.024 19732931 PMC2915564

[B156] Soumahoro M. K. Gérardin P. Boëlle P. Y. Perrau J. Fianu A. Pouchot J.s. (2009). Impact of Chikungunya virus infection on health status and quality of life: a retrospective cohort study. PLoS ONE 4:e7800. 10.1371/journal.pone.0007800 19911058 PMC2771894

[B157] Sourisseau M. Schilte C. Casartelli N. Trouillet C. Guivel-Benhassine F. Rudnicka D.. (2007). Characterization of reemerging Chikungunya virus. PLoS Pathog. 3:e89. 10.1371/journal.ppat.0030089 17604450 PMC1904475

[B158] Subudhi B. B. Chattopadhyay S. Mishra P. Kumar A. (2018). Current strategies for inhibition of Chikungunya infection. Viruses 10:E235. 10.3390/v10050235 29751486 PMC5977228

[B159] Sudeep A. B. Parashar D. (2008). Chikungunya: an overview. J. Biosci. 33, 443–449. 10.1007/s12038-008-0063-2 19208970

[B160] Tanabe E. L. L. Tanabe I. S. B. Santos E. C. D. Marques J. P. D. S. Borges A. A. Lima M. C.. (2018). Report of East-Central South African Chikungunya virus genotype during the 2016 outbreak in the Alagoas State, Brazil. Rev. Inst. Med. Trop. Sao Paulo 60, e19. 10.1590/s1678-9946201860019 29694603 PMC5956549

[B161] Teng T. S. Kam Y. W. Lee B. Hapuarachchi H. C. Wimal A. Ng L. C.. (2015). A systematic meta-analysis of immune signatures in patients with acute Chikungunya virus infection. J. Infect. Dis. 211, 1925–1935. 10.1093/infdis/jiv049 25635123 PMC4442625

[B162] Teo T. H. Chan Y. H. Lee W. W. Lum F. M. Amrun S. N. Her Z.. (2017). Fingolimod treatment abrogates chikungunya virus-induced arthralgia. Sci. Transl. Med. 9:eaal1333. 10.1126/scitranslmed.aal1333 28148838

[B163] Teo T. H. Her Z. Tan J. J. Lum F. M. Lee W. W. Chan Y. H.. (2015). Caribbean and La Reunion Chikungunya virus isolates differ in their capacity to induce proinflammatory Th1 and NK cell responses and acute joint pathology. J. Virol. 89, 7955–7969. 10.1128/JVI.00909-15 25995257 PMC4505608

[B164] Teo T. H. Lum F. M. Claser C. Lulla V. Lulla A. Merits A.. (2013). A pathogenic role for CD4^+^ T cells during Chikungunya virus infection in mice. J. Immunol. 190, 259–269. 10.4049/jimmunol.1202177 23209328

[B165] Thanapati S. Ganu M. A. Tripathy A. S. (2017). Differential inhibitory and activating NK cell receptor levels and NK/NKT-like cell functionality in chronic and recovered stages of chikungunya. PLoS ONE 12:e0188342. 10.1371/journal.pone.0188342 29182664 PMC5705157

[B166] Thangamani S. Higgs S. Ziegler S. Vanlandingham D. Tesh R. Wikel S. (2010). Host immune response to mosquito-transmitted chikungunya virus differs from that elicited by needle inoculated virus. PLoS ONE 5:e12137. 10.1371/journal.pone.0012137 20711354 PMC2920837

[B167] Thiberville S. D. Boisson V. Gaudart J. Simon F. Flahault A. de Lamballerie X. (2013a). Chikungunya fever: a clinical and virological investigation of outpatients on Reunion Island, South-West Indian Ocean. PLoS Negl. Trop. Dis. 7:e2004. 10.1371/journal.pntd.0002004 23350006 PMC3547841

[B168] Thiberville S. D. Moyen N. Dupuis-Maguiraga L. Nougairede A. Gould E. A. Roques P.. (2013b). Chikungunya fever: epidemiology, clinical syndrome, pathogenesis and therapy. Antiviral Res. 99, 345–370. 10.1016/j.antiviral.2013.06.009 23811281 PMC7114207

[B169] Tiwari M. Parida M. Santhosh S. R. Khan M. Dash P. K. Rao P. V. (2009). Assessment of immunogenic potential of Vero adapted formalin inactivated vaccine derived from novel ECSA genotype of Chikungunya virus. Vaccine 27, 2513–2522. 10.1016/j.vaccine.2009.02.062 19368794

[B170] Torres J. R. Falleiros-Arlant L. H. Dueñas L. Pleitez-Navarrete J. Salgado D. M. Castillo J. B. (2016). Congenital and perinatal complications of chikungunya fever: a Latin American experience. Int. J. Infect. Dis. 51, 85–88. 10.1016/j.ijid.2016.09.009 27619845

[B171] Tretyakova I. Hearn J. Wang E. Weaver S. Pushko P. (2014). DNA vaccine initiates replication of live attenuated chikungunya virus *in vitro* and elicits protective immune response in mice. J. Infect. Dis. 209, 1882–1890. 10.1093/infdis/jiu114 24585894 PMC4038148

[B172] Tsetsarkin K. A. Chen R. Leal G. Forrester N. Higgs S. Huang J.. (2011). Chikungunya virus emergence is constrained in Asia by lineage-specific adaptive landscapes. Proc. Natl. Acad. Sci. U.S.A. 108, 7872–7877. 10.1073/pnas.1018344108 21518887 PMC3093459

[B173] Tsetsarkin K. A. Chen R. Weaver S. C. (2016). Interspecies transmission and chikungunya virus emergence. Curr. Opin. Virol. 16, 143–150. 10.1016/j.coviro.2016.02.007 26986235 PMC4824623

[B174] Tsetsarkin K. A. Vanlandingham D. L. McGee C. E. Higgs S. (2007). A single mutation in chikungunya virus affects vector specificity and epidemic potential. PLoS Pathog. 3:e201. 10.1371/journal.ppat.0030201 18069894 PMC2134949

[B175] Venugopalan A. Ghorpade R. P. Chopra A. (2014). Cytokines in acute chikungunya. PLoS ONE 9:e111305. 10.1371/journal.pone.0111305 25343623 PMC4208842

[B176] Voss J. E. Vaney M. C. Duquerroy S. Vonrhein C. Girard-Blanc C. Crublet E.. (2010). Glycoprotein organization of Chikungunya virus particles revealed by X-ray crystallography. Nature 468, 709–712. 10.1038/nature09555 21124458

[B177] Wahid B. Ali A. Rafique S. Idrees M. (2017). Global expansion of chikungunya virus: mapping the 64-year history. Int. J. Infect. Dis. 58, 69–76. 10.1016/j.ijid.2017.03.006 28288924

[B178] Wang D. Suhrbier A. Penn-Nicholson A. Woraratanadharm J. Gardner J. Luo M.. (2011a). A complex adenovirus vaccine against chikungunya virus provides complete protection against viraemia and arthritis. Vaccine 29, 2803–2809. 10.1016/j.vaccine.2011.01.108 21320541 PMC3061842

[B179] Wang E. Kim D. Y. Weaver S. C. Frolov I. (2011b). Chimeric Chikungunya viruses are nonpathogenic in highly sensitive mouse models but efficiently induce a protective immune response. J. Virol. 85, 9249–9252. 10.1128/JVI.00844-11 21697494 PMC3165793

[B180] Wauquier N. Becquart P. Nkoghe D. Padilla C. Ndjoyi-Mbiguino A. Leroy E. M. (2011). The acute phase of Chikungunya virus infection in humans is associated with strong innate immunity and T CD8 cell activation. J. Infect. Dis. 204, 115–123. 10.1093/infdis/jiq006 21628665 PMC3307152

[B181] Weaver S. C. (2013). Urbanization and geographic expansion of zoonotic arboviral diseases: mechanisms and potential strategies for prevention. Trends Microbiol. 21, 360–363. 10.1016/j.tim.2013.03.003 23910545 PMC5193003

[B182] Weber C. Büchner S. M. Schnierle B. S. (2015). A small antigenic determinant of the Chikungunya virus E2 protein is sufficient to induce neutralizing antibodies which are partially protective in mice. PLoS Negl. Trop. Dis. 9:e0003684. 10.1371/journal.pntd.0003684 25905779 PMC4407984

[B183] Weger-Lucarelli J. Aliota M. T. Kamlangdee A. Osorio J. E. (2015). Identifying the role of E2 domains on alphavirus neutralization and protective immune responses. PLoS Negl. Trop. Dis. 9:e0004163. 10.1371/journal.pntd.0004163 26473963 PMC4608762

[B184] Weger-Lucarelli J. Chu H. Aliota M. T. Partidos C. D. Osorio J. E. (2014). A novel MVA vectored Chikungunya virus vaccine elicits protective immunity in mice. PLoS Negl. Trop. Dis. 8:e2970. 10.1371/journal.pntd.0002970 25058320 PMC4109897

[B185] Wilson J. A. Prow N. A. Schroder W. A. Ellis J. J. Cumming H. E. Gearing L. J.. (2017). RNA-Seq analysis of chikungunya virus infection and identification of granzyme A as a major promoter of arthritic inflammation. PLoS Pathog. 13:e1006155. 10.1371/journal.ppat.1006155 28207896 PMC5312928

[B186] Zammarchi L. Fortuna C. Venturi G. Rinaldi F. Capobianco T. Remoli M. E.. (2016). Recent Chikungunya virus infection in 2 travelers returning from Mogadishu, Somalia, to Italy, 2016. Emerg. Infect. Dis. 22. 10.3201/eid2211.161225 27513985 PMC5088032

[B187] Ziegler S. A. Lu L. da Rosa A. P. Xiao S. Y. Tesh R. B. (2008). An animal model for studying the pathogenesis of chikungunya virus infection. Am. J. Trop. Med. Hyg. 79, 133–139. 18606777

